# Optical phantoms for the characterization and validation of imaging and spectroscopy: a review

**DOI:** 10.1117/1.BIOS.3.2.022511

**Published:** 2026-06-04

**Authors:** Vivian C. Krause, Alec B. Walter, Anita Mahadevan-Jansen, E. Duco Jansen

**Affiliations:** a Vanderbilt University, Department of Biomedical Engineering, Nashville, Tennessee, United States; b Vanderbilt University, Vanderbilt Biophotonics Center, Nashville, Tennessee, United States; c Vanderbilt University Medical Center, Department of Neurosurgery, Nashville, Tennessee, United States; d Vanderbilt University Medical Center, Department of Surgery, Nashville, Tennessee, United States; e Vanderbilt University Medical Center, Department of Otolaryngology, Nashville, Tennessee, United States

**Keywords:** optical phantoms, tissue optics, skin tone, imaging, spectroscopy, characterization

## Abstract

**Significance:**

Tissue-mimicking optical phantoms are an essential tool in the development, characterization, and calibration of instruments used in optical imaging, spectroscopy, diagnosis, and treatment of disease.

**Aim:**

Major developments in optical phantoms have been enabled by recent advances in material development and characterization, as well as sophisticated manufacturing techniques. In this review, we will focus primarily on the developments in the field over the past 20 years since the seminal review paper on this topic by Pogue et al. in 2006. Throughout, we have attempted to provide the reader with practical guidelines to consider when producing phantoms.

**Approach:**

Here, we review and assess phantom geometries and available materials for phantom matrices, as well as absorbing and scattering agents. We then review and discuss the use of tissue phantoms in a variety of optical imaging and spectroscopic modalities and address the use and challenges of models of light propagation to extract optical properties from the measured physical parameters. Finally, we highlight the use and utility of tissue optical phantoms applied to the specific challenge of accounting for melanin when using optical systems to extract optical properties and by extension, physiological information, through skin.

**Results:**

In this paper, we provide a comprehensive review of material selection and fabrication considerations for the design of tissue optical phantoms. We then describe how tissue optical phantoms are designed and used for specific optical modalities, emphasizing both recent developments and current limitations, and making the argument for more standardization and detailed reporting of tissue phantom production.

**Conclusions:**

Throughout this review, we provide the reader with practical “how to” information for the production of tissue phantoms that are targeted for the optical modality and characterization goal. As innovative optical imaging and spectroscopy technologies are continually developed, rigorous testing and validation with well-designed optical phantoms are necessary.

Statement of Discovery There is a growing need for standardization in phantom design as phantom testing becomes an increasingly critical component in the development and regulatory practices of optical technologies. This work reviews the current state of tissue phantoms for the field of optical imaging and spectroscopy, providing practical information on sample geometry, materials, and modality-specific requirements to inform phantom design.

## 1 Introduction

In biomedical research, tissue-mimicking phantoms are essential for the development, characterization, and calibration of a diverse array of instruments in imaging and spectroscopy, especially when used for clinical applications, such as the diagnosis and treatment of disease. These so-called "tissue phantoms" are designed and produced to mimic one or more physical and/or biochemical characteristics of biological tissue in a controlled and reproducible way. Although tissue phantoms are routinely used for various medical devices or imaging modalities, including magnetic resonance imaging (MRI), computed tomography (CT), ultrasound (US), and others, this review focuses specifically on optical tissue phantoms where the optical properties of the phantom, namely, absorption, scattering, and refractive index, as well as secondary sources of optical activity such as fluorescence, polarization, or inelastic scattering (Raman scattering), are carefully controlled depending on the application. Regulatory bodies recommend or may require the use of standardized tissue phantoms for good manufacturing practices, system characterization for metrics such as spatial resolution, spectral resolution, signal-to-noise characteristics, calibration, and the assessment of system performance.^1^ Importantly, the use of tissue optical phantoms enables the comparison of device performance over time at a given location and between users, laboratories, or clinics.

**Fig. 1:**
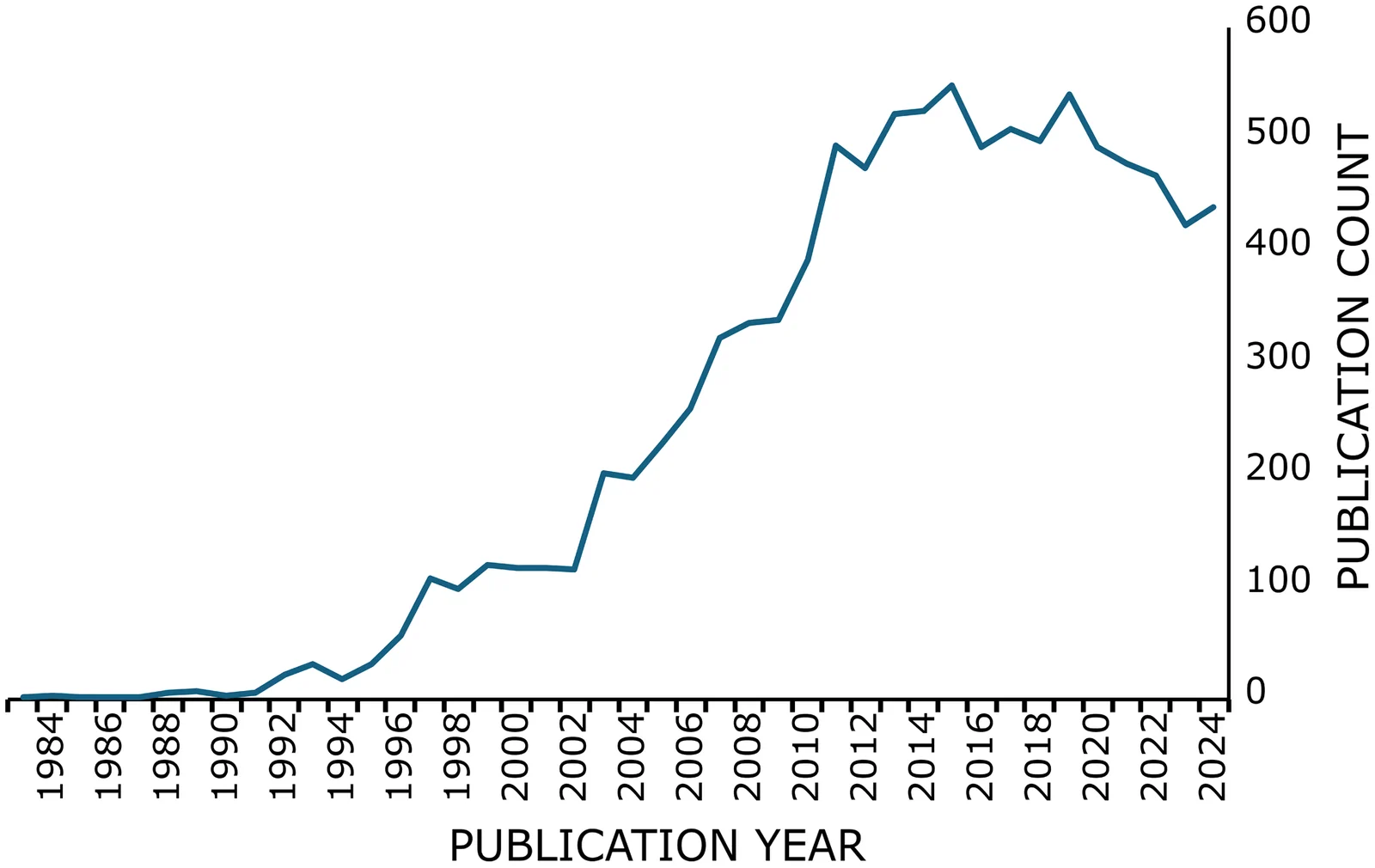
Bibliometric search quantifying the number of publications on the topic of optical tissue phantoms published since 1980. The search was completed on Web of Science with the query of papers published with titles containing “optical tissue phantoms OR optical phantoms OR optical tissue-mimicking phantoms OR optical tissue-mimicking materials” between the dates of January 1, 1980, and December 31, 2024.

Numerous papers have been published on tissue optical phantoms over the past four and a half decades. A bibliometric search for published papers with titles containing “optical tissue phantoms OR optical phantoms OR optical tissue-mimicking phantoms OR optical tissue-mimicking materials” yielded 10,521 publications between 1980 and 2024, with over 400 being published each year since 2012, as demonstrated in Fig. 1. Additional publications, beyond those included in Fig. 1, present novel optical approaches that use phantoms to validate their performance without explicitly including the term “phantoms” in their title. Although the vast number of applications and innovations in the optical phantom space precludes a comprehensive review of all possible use cases of tissue optical phantoms, Table 1 summarizes focused reviews that describe the development and/or use of tissue optical phantoms used in specific optical modalities.

**Table 1:** Relevant review papers that describe the development and/or use of tissue optical phantoms used in specific optical modalities.

Authors	Modality	Year of publication	Citation
Pogue and Patterson	General phantom development	2006	2
Lamouche et al.	Optical coherence tomography	2012	3
Dabbagh et al.	Thermal therapy modalities	2014	4
Yazdi et al.	Particle image velocimetry	2018	5
Chue-Sang et al.	Polarimetry	2019	6
Vardaki and Kourkoumelis	Raman spectroscopy	2020	7
Hacker et al.	General phantom development	2022	8
Dinh et al.	Fluorescence imaging, surgical guidance	2022	9
Christie et al.	Photoacoustic imaging	2023	10
Kwon et al.	Optical coherence tomography, photoacoustic imaging, digital holographic microscopy, optical diffraction tomography, oximetry	2024	11
Kulmaganbetov	Optical coherence tomography	2025	12

**Fig. 2:**
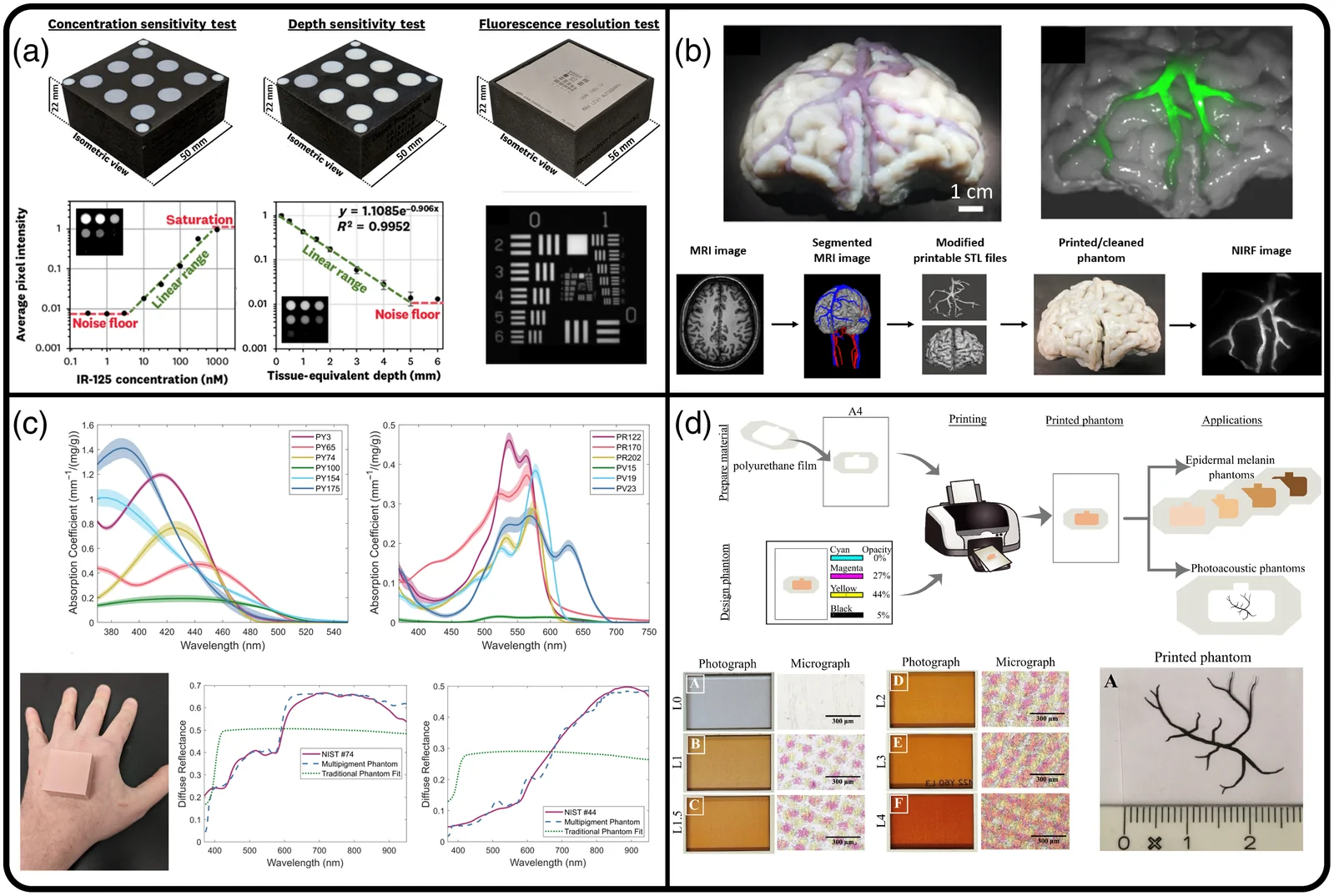
Examples highlighting recent developments and modern techniques for optical phantoms. (a) Phantoms designed with patterned fluorescent material can be used to characterize multiple performance metrics of fluorescence imaging systems, including limit of detection, depth sensitivity, and resolution. Adapted with permission from Ref. 13. (b) 3D printing advances have enabled the production of anatomically complex optical phantoms directly from medical imaging data. Adapted with permission from Ref. 14 © Optica Publishing Group. (c) Multipigment phantoms combine the absorption and scattering properties of multiple discrete pigments to achieve homogeneous, skin-mimicking diffuse reflectance phantoms. Adapted with permission from Ref. 15. (d) Thin epidermal phantoms with variable skin tone and biomimicking blood vessel patterns can be made using a commercial laser printer to deposit colored toner onto polyurethane film. Adapted with permission from Ref. 16 © Optica Publishing Group.

An outstanding comprehensive review paper on tissue optical phantoms was published by Pogue et al. nearly 20 years ago.^2^ With the continuous development of materials, improved understanding of tissue optics, and the advent and advancement of sophisticated production techniques such as 3D printing, the field of tissue phantoms has made tremendous strides in recent years. Furthermore, improvements in efficiency, sensitivity, and cost of instrumentation, especially light sources and detectors that can measure tissue optical properties both directly and indirectly, necessitate that we revisit the state of the art in tissue optical phantoms in 2025. Major advances over the past two decades include the development of realistic, broad-spectrum phantoms that accurately replicate tissue optical properties over a wide range of wavelengths, advanced two- and three-dimensional geometries enabled by recent developments in 3D printing, and advanced machining, and additive manufacturing methodologies. These recent developments in the field of optical phantoms are summarized in Fig. 2. Moreover, there have been significant advances in our understanding of light transport in tissue (especially in the sub-diffuse regime), which lie at the basis of the computational models that are used to extract optical properties from the physical measurements made with phantoms.

With any phantom, it is important to intentionally tailor the phantom design to the question to be asked and/or the metric to be determined. The design of a tissue optical phantom can range from a simple liquid or gel-like object with homogeneous optical properties accurate to a tissue of interest at a specified wavelength, to a complex 3D tissue-mimicking phantom that reproduces broadband spectral behavior.^14^^,^^17^^–^^20^ Recent advances in fabrication have enabled anatomically realistic representations of tissue layers and structures (e.g., blood vessels, tumors), with realistic optical properties over a wide spectral range, and intricate features such as (micro) channels, which may allow for flow of blood, blood-like liquids, or optical contrast agents.^14^^,^^19^^–^^22^ Nevertheless, phantom geometry must be chosen wisely: simple, well-controlled designs often enable the most robust and reliable tests for optical systems, and geometric complexity should be introduced only as it is relevant to the intended evaluation. The major categories of phantom geometries are described in Sec. 2. Beyond geometry, material selection is equally critical in phantom design and typically depends on the desired optical and physical properties of the phantom. The three main categories of components that determine the characteristics of a tissue optical phantom include: (1) a matrix material, which is the main constituent of the phantom that provides structure and serves as the base for the other components of the phantom; (2) a scatterer, which contributes the desired scattering behavior of the target tissue; and (3) an absorber, which contributes the desired absorption behavior of the target tissue. The most common types of matrix materials, scatterers, and absorbers, and their tradeoffs in phantom design are discussed in Sec. 3. It should be noted that in addition to these three categories, other constituents may be added in specific applications for purposes of stability, antibacterial treatment, or achieving certain physical properties. With a wide choice of matrix materials, wavelength-dependent absorbers and scatterers, optically active agents (fluorophores or Raman active), and phantom geometries, the researcher must have a thorough understanding of the choices available to them, the advantages and limitations of each, the effect of their choices on temporal and spatial stability of the phantom (i.e., shelf live and photostability), the optical measurement they intend to make, and the quantitative metrics and properties they hope to extract from the phantom experiment. In our current review, we will capture the current state of manufacturing phantoms by reviewing the phantom designs, materials, and manufacturing methods that have been most commonly utilized over the past two decades since the seminal review paper by Pogue.

In addition to material and manufacturing considerations, one must consider that tissue optical phantoms are used in a broad range of optical technologies that employ a wide range of different optical contrast modalities for clinical and preclinical use. These include, but are not limited to, techniques using diffuse optics, such as hyperspectral imaging and pulse oximetry, optical coherence tomography (OCT), steady state and time-resolved fluorescence imaging and spectroscopy (both autofluorescence as well as targeted exogenous fluorescence labels for molecular imaging), Raman spectroscopy (RS), photoacoustic imaging (PAI) and tomography (PAT), and a variety of biomedical systems that use one or more of these modalities for clinical applications. Given that these modalities use different contrast mechanisms to characterize and differentiate tissues, it stands to reason that a single standard optical tissue phantom will not be sufficient, and instead, modality-specific tissue phantoms are necessary. Thus, in Sec. 4, we provide examples of the considerations for optical phantoms for a variety of optical modalities, including diffuse optics, fluorescence imaging, PAI, OCT, and RS. In Sec. 5, we provide an overview of the practical considerations that should inform the design and implementation of phantoms in the field of biophotonics, with particular attention to factors influencing reproducibility. This is followed by Sec. 6, which utilizes an example phantom use case to explore how the information and considerations discussed in the preceding section can be applied.

Overall, the objective of this review is to provide the reader with an overview of recent developments in the field of tissue optical phantoms and with a practical guide on the considerations that go into the design and production of useful and reproducible tissue optical phantoms that can be used in the characterization and calibration of optical imaging and spectroscopic instruments that are used for the screening, diagnosis, and treatment of disease. A timely and unique use case example, demonstrating the powerful impact that tissue phantoms can have on ensuring that accurate physiological parameters are extracted from optical measurements, is described by considering the effect of skin tone (e.g., melanin concentration) on optical measurements.

### 1.1 Standardization Efforts for Optical Tissue Phantoms

In contrast to fields such as MRI and ultrasound, where community consensus and regulation surrounding phantom use are currently employed,^23^^–^^25^ biophotonics has not yet reached a comparable level of consensus, although efforts toward reproducibility and standardization are ongoing.^26^^–^^29^ While phantoms should be tailored to the use case and the requirements of the optical modality being employed, there is heterogeneity in the designs and materials used within a modality to characterize or test the same metrics across different research groups. One factor that limits the standardization of optical phantoms is the widespread use of custom phantoms developed by individual laboratories and companies to meet the specific requirements of their instrumentation. Although these designs are often described in the literature, enabling a degree of reproducibility across groups, there remains no broad consensus on the appropriate phantom design, materials, or performance criteria for a given validation task. Another factor that impedes progress toward standardization of optical phantoms for the biophotonics community is insufficient detail in the reporting of phantom designs and manufacturing methods, which limits reproducibility across groups. To address this, in Sec. 5.3, we provide recommendations for phantom reporting aimed at improving reproducibility and enabling more consistent comparisons and testing of systems across groups. Finally, due to the specific requirements of phantoms used for a given optical modality, it is difficult to make generally applicable recommendations for standard phantoms across biophotonics. Therefore, we suggest that standardization should be specific to tissue optical phantoms used for a given imaging or spectroscopy modality.

An exemplary effort towards expert consensus on phantom use is the photoacoustic community, which has established the International Photoacoustic Standardization Consortium (IPASC). This consortium aims to establish standardized test methods, data handling practices, and consensus on data formats to improve the quality and reproducibility of photoacoustic imaging (PAI) research, and improve the transition of photoacoustic technologies to clinical applications.^30^ Unsurprisingly, one of the key aspects of the IPASC standards includes guidelines for effective and consistent phantom design, thus far focusing on material properties that are essential for consistently evaluating imaging quality metrics, such as spatial resolution and imaging depth. Although the IPASC standards are an ongoing development, they demonstrate promise and can help guide the standardization of phantoms for other optical modalities.

Multiple government agencies and professional organizations have also begun to exert influence on the standardization of optical phantoms in recent years. In an effort to develop best practices and methodologies for phantom manufacturing and potentially establish accepted standards for tissue optical phantoms, the National Science Foundation, in collaboration with the National Institutes of Health (NIH) and the FDA in the United States, has established an Industry-University Cooperative Research Center focused on optical tissue-mimicking phantoms for biomedical applications. In addition, the FDA’s Center for Devices and Radiological Health maintains the Regulatory Science Tools Catalog, a repository of regulatory tools, including phantoms, previously developed by its own scientists.^31^ In a similar effort, the FDA, National Institute of Standards and Testing (NIST), and the National Institute of Biomedical Imaging and Bioengineering (NIBIB) support the National Medical Phantom Library (NMPL) joint effort, which aims to create centralized access to imaging phantoms for a wide variety of medical imaging modalities. This effort consists of two main sites, NMPL NIST, which has been in operation since 2020 and focus mostly on MRI phantoms, and NMPL NIBIB, a recently started endeavor focusing on other imaging modalities with a major focus on optical techniques.^32^^,^^33^ As the NMPL expands to include phantoms for various modalities in biophotonics, it is important that phantom developers and phantom users provide support for this resource to accelerate widespread effects on standardization. These efforts toward standardization of phantoms in the field of biophotonics demonstrate promise and will pave the way for innovative optical designs and collaboration across groups.

### 1.2 Commercial Activities for Optical Tissue Phantoms

In tandem with the development and commercialization of innovative optical devices for biomedical applications, the rigorous testing of these systems is imperative. Phantoms can be used for validation under different physiologically relevant conditions such as blood flow and oxygenation, anatomical shapes, sizes, and compositions of tissue and tissue structures, varied tissue compositions, and broad tissue optical properties, including skin pigmentation. Unlike other imaging modalities, such as MRI or ultrasound, where commercially available phantoms are now commonly used for standardization and inter-system comparisons, optical technologies are typically tested with single-use, in-house optical phantoms, or custom-designed phantoms obtained from specialized vendors, which assess specific characteristics of the system that a company or lab is developing. At the present time, only two companies that specifically develop and sell optical phantoms in the marketplace exist: QUEL Imaging (Vermont, United States) and BioPixS (Cork, Ireland).^34^^,^^35^ In both cases, these commercial entities were spun off from innovative tissue optical phantom research that originated in academic research labs.

Because validation tools for optical and medical device companies often rely on custom-designed phantoms provided by specialized vendors, as well as those licensed from academic laboratories or developed internally, they are frequently application-specific and not widely shared or commercially available across the field.^34^^–^^37^ This practice can hinder the establishment of consistent validation and calibration practices across the field.

Current commercial activities in optical phantom development and manufacturing include (a) optical material development (novel matrix materials, development of novel absorbers, fluorophores, and scatterers across the optical spectrum), (b) characterization of optical phantoms (establishing "ground truth" optical properties), and (c) development of complex geometries, including anatomically realistic phantoms (sometimes referred to as anthropomorphic phantoms) that can mimic specific tissue structures or anatomical features. Optical phantoms for specific imaging modalities are commercially available for optical coherence tomography, fluorescence imaging, diffuse optical imaging, and functional near-infrared spectroscopy.^34^^,^^35^ Additional recent developments include dynamic phantoms that have embedded flow channels through which optical contrast agents, such as fluorescent molecules or blood, can flow.^38^ Looking to the future, the availability of reproducible, well-characterized commercial phantoms demonstrates a strong potential to play a key role in community consensus on phantoms to use for specific tasks.

## 2 Phantom Geometry

**Fig. 3:**
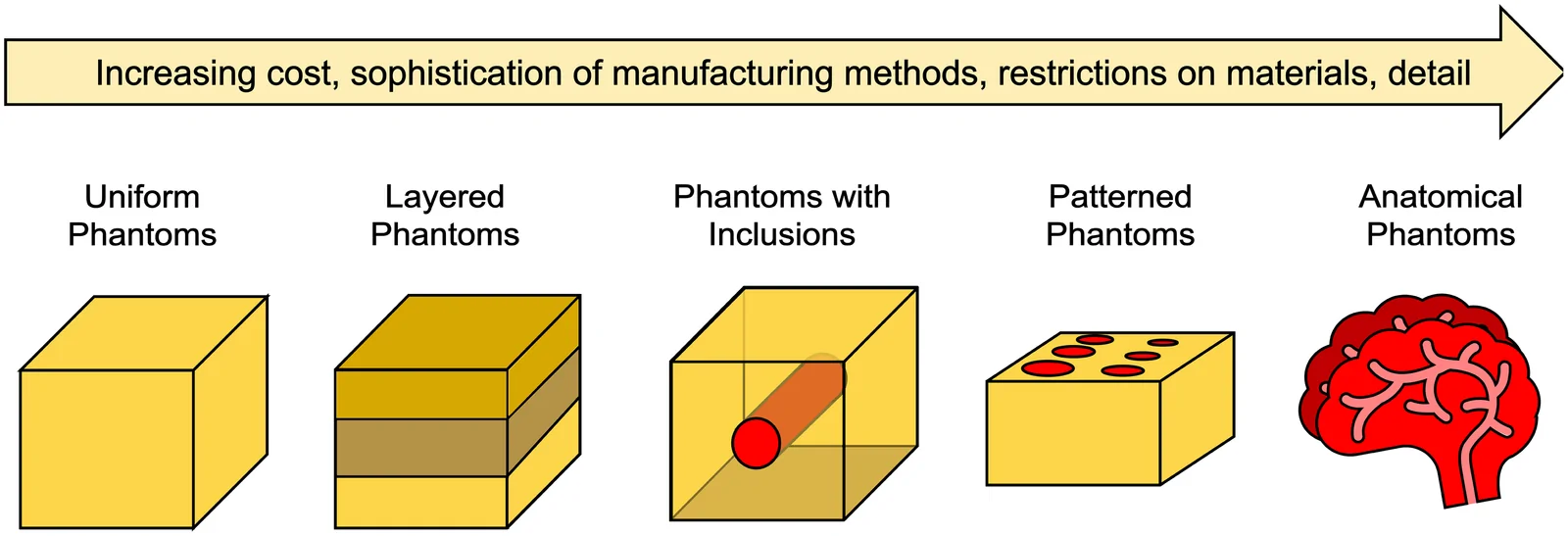
Schematic representation of five common phantom geometry categories and summary of their characteristic tradeoffs.

Tissue optical phantoms can be constructed in a variety of geometries to mimic the structure and optical properties of biological tissues. The choice of phantom geometry depends on the specific optical experiment and tissue being modeled, as well as the scientific question to be addressed by performing the phantom experiment. In general, the goal should be to use the simplest phantom possible for the research question at hand. Figure 3 summarizes the five main categories of phantom geometries that are typically used: uniform phantoms, layered phantoms, phantoms with inclusions, patterned phantoms, and anatomically realistic phantoms. Phantoms may contain features from one or multiple categories.

Uniform phantoms are typically made by homogeneously mixing optical absorbers and scatterers into a transparent matrix material.^15^^,^^39^^–^^42^ Uniform phantoms with homogeneous optical properties provide a simple model for baseline measurements and for measurements where the average, bulk optical properties of the tissue are sufficient for the problem at hand. However, they lack the structural complexity of real tissues, which is required to comprehensively and accurately assess some optical techniques. The advantage of uniform phantoms is their simplicity and the fact that they are relatively easy to manufacture and accurately reproduce.

Phantoms consisting of two or more layers with distinct optical properties can represent the stratified structure of skin or other layered tissues.^18^^,^^21^^,^^43^^–^^50^ Layered phantoms can be fabricated by stacking or molding separate layers with distinct optical properties. The challenge is in ensuring good optical coupling between the layers to avoid creating air gaps, which will result in high specular reflections. In general, layered and patterned phantoms offer more realistic modeling of layered tissue architecture, with distinct properties in each layer, such as the retina or skin.

Phantoms with one or multiple inclusions can represent the optical effects of localized structures such as blood vessels, tumors, or fluorescent markers.^14^^,^^19^^,^^21^^,^^45^^,^^46^^,^^49^^–^^59^ These types of phantoms contain an embedded localized region with distinct optical properties, such as a highly absorbing or fluorescent region. Inclusions can be made by selectively doping part of the phantom matrix or by physically embedding an object with different optical properties within the bulk phantom material at a specified depth. The limitation is that the inclusion geometry is often simple, such as a sphere or cylinder, rather than anatomically accurate objects. Phantoms with inclusions are useful for studying light propagation in tissues that contain structures with optical properties that differ significantly from the surrounding bulk tissue, but typically provide approximate behavior due to the inclusions rather than ground truth.

Patterned phantoms refer to more complex versions of inclusion phantoms that have multiple inclusions with a range of properties to meet specific validation and/ or characterization goals.^13^^,^^22^^,^^52^^,^^59^^–^^69^ This category includes inclusions at various depths in the phantom, such as vessel-mimicking structures with multiple radii. An increasingly prevalent type of patterned phantom is a composite phantom, which contains an array of different structures designed to enable the characterization of multiple system performance parameters using only a single phantom, as shown in Fig. 2(a).^13^^,^^52^^,^^59^^,^^63^^–^^68^ Although patterned phantoms are complex in structure and require additional manufacturing considerations, they are not geometrically biomimicking or anatomically realistic.

Anatomical phantoms, which often contain the most complex geometries, are those with 3D structures that directly mimic the size and shape of one or multiple tissue structures, such as blood vessel patterns.^14^^,^^17^^–^^20^^,^^44^^,^^56^^,^^71^^–^^75^ While more realistic, they require sophisticated fabrication methods and may require high-resolution imaging methods, such as MRI, to map the 3D structures of interest, as shown in Fig. 2(b).^14^^,^^71^^,^^72^ These anatomical phantoms can be fabricated by techniques such as 3D printing, casting around physical templates, or selective photopolymerization.^14^^,^^17^^–^^20^^,^^44^^,^^56^^,^^69^^,^^71^^–^^75^ Although these novel designs allow more accurate optical properties, they are not necessary for all optical testing applications.

## 3 Phantom Components

### 3.1 Matrix Materials

**Fig. 4:**
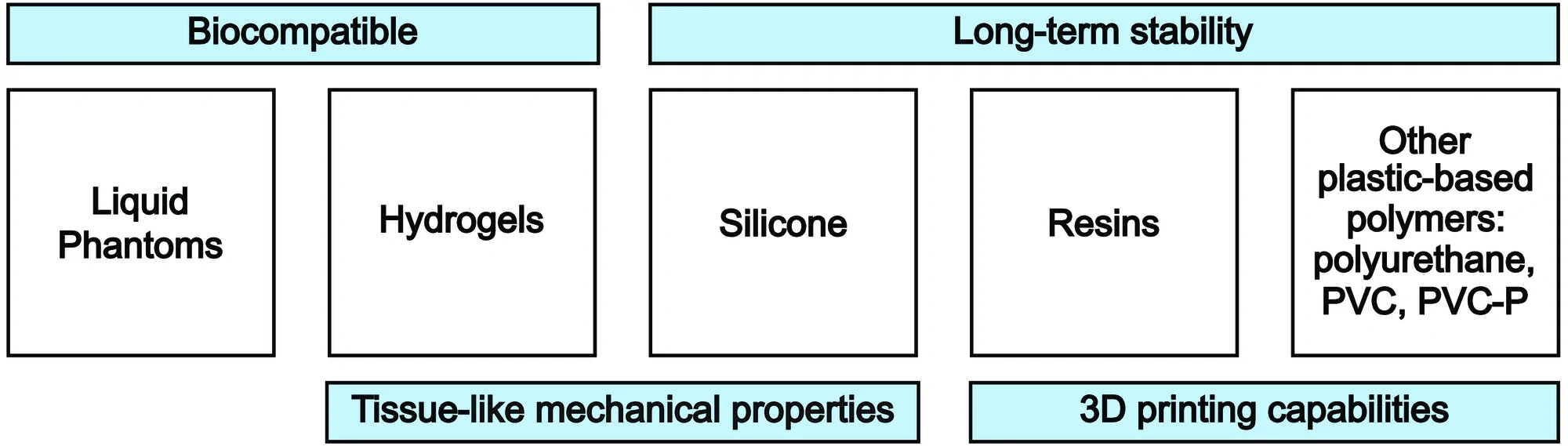
Summary of common matrix materials and their main tradeoffs to inform phantom material selection.

The matrix material is the base component that provides shape and structure to the optical phantom and contains the other constituents that provide the desired optical behavior. However, matrix materials themselves have intrinsic optical [including absorption, scattering, and refractive index at the wavelength(s) of interest], mechanical, and acoustic properties that impact the overall characteristics of the optical phantom that must be carefully considered when selecting a matrix material. In addition, factors associated with ease of use, desired geometry of the phantom, long-term stability (i.e., shelf life of the phantom), cost, and manufacturing requirements must also be considered. Although a wide variety of materials have been used as optical phantom matrices, this review will cover the properties and constraints associated with the most commonly utilized materials: liquid solutions, hydrogels, silicone, resins, and other common plastic-based materials, as summarized in Fig. 4.

#### 3.1.1 Liquid phantoms

Solutions or liquid suspensions of one or more components, such as specific absorbing and scattering agents, are the simplest models for reference phantoms. Liquid phantoms are usually aqueous, relying on water (pure or as PBS) for their matrix material as it allows for broad compatibility with a range of other components, such as biological substances.^57^^,^^76^^–^^81^ However, other liquid solvents, such as DMSO and ethanol, can be used as the base for specific applications where water is not compatible with one or more other phantom materials, such as a fluorophore that may require an organic solvent.^82^^–^^84^ In their simplest form, liquid phantoms contain only a single scatterer or absorber.^42^^,^^84^^–^^86^ Intralipid and India ink together form one of the most common combinations of a single absorber and scatter used in liquid phantoms, with their optical properties having been extensively characterized.^87^

Although liquid phantoms are quick to produce, cost-efficient in comparison to other phantom designs and can provide important insight, they have significant limitations. In particular, their shelf life is limited to single use or repeated use over a short period of time. Furthermore, when the liquid phantom includes suspended particles, these may either settle to the bottom or float to the surface of the liquid. This results in the phantom having inhomogeneous optical properties that change throughout the duration of its use, though periodic homogenization can mitigate these effects. Finally, liquid phantoms are limited in the geometries they can take without the use of other materials, being unable to form layered arrangements, complex shapes, or anatomical morphologies. They are, however, commonly used as the liquid component in more geometrically complex flow-based optical phantoms.^88^ Despite these limitations and challenges, liquid phantoms are a practical and useful tool for the preliminary testing and characterization of optical systems.

#### 3.1.2 Hydrogels

Hydrogels are materials with an aqueous base and have a given structure through a flexible network of polymer chains. These materials, including agar, polyacrylamide, polyvinyl alcohol, and, most commonly, gelatin, are widely used in optical phantoms due to their unique blend of tissue-mimicking physical, mechanical, and optical properties.^3^^,^^9^^,^^10^^,^^18^^,^^40^^,^^41^^,^^47^^,^^50^^,^^52^^–^^54^^,^^56^^,^^58^^,^^68^^,^^88^^–^^112^ Contrary to most other matrix materials, hydrogels allow for flexible phantoms that can more closely approximate the mechanical characteristics of soft biological tissue.^89^^,^^105^ These flexible materials can also be easily shaped and cast into uniform phantoms, inclusion phantoms, or thin multilayered phantoms.^89^^,^^100^^,^^108^ Furthermore, hydrogels are biocompatible, which allows biological components, such as blood, fat, and organic fluorophores, to be easily incorporated into the phantom. Such additions make hydrogels serve as ideal matrix materials for making phantoms with tissue-like properties.^3^^,^^100^^–^^103^^,^^108^ Hydrogels are typically optically transparent in the visible and near-infrared spectral range and have an intrinsic refractive index of ∼1.35, which is close to that of soft tissues.^89^^,^^104^^,^^105^ In addition, tuning the water content of hydrogels provides control over the concentration-dependent optical properties of the material.^3^^,^^89^^,^^100^^,^^105^ The mechanical properties of hydrogels, such as the speed of sound, Young’s modulus, acoustic attenuation, and impedance, can also be controlled through water content, which is beneficial for photoacoustic applications, laser ablation, or studies involving elastography.^10^^,^^88^^,^^90^^,^^91^^,^^113^^,^^114^

Though hydrogels have properties that are useful for phantom development, there are many factors to consider when working with these materials. One of the biggest issues when utilizing hydrogels is their stability over time. Due to their high water content, hydrogel phantoms are prone to dehydration, especially during use, which can alter both their mechanical and optical properties, especially at the outer surfaces of the phantom. In addition, when hydrogels are used in structured or layered phantoms, diffusion of water and dyes between layers has been observed, also limiting their use time.^9^^,^^111^ Furthermore, the geometric complexity possible with hydrogel phantoms is limited due to their aqueous base and structural instability.^100^ Various strategies have been developed to mitigate these challenges, including refrigeration, air-tight storage, and the inclusion of additives that enhance water retention, improve physical stability, inhibit bacterial growth, and otherwise help to maintain stable optical properties over time.^99^^,^^112^ Under optimal storage conditions, hydrogel phantoms have been reported to last over 2 years.^106^

#### 3.1.3 Silicone

Silicone is another widespread matrix material for tissue-mimicking phantoms, which provides a durable and flexible phantom base.^9^^,^^21^^,^^43^^,^^46^^,^^115^^–^^126^ The most common silicone material for optical phantoms is polydimethylsiloxane (PDMS). Silicone can be molded into custom, complex geometries to mimic biological structures and can be used as a base material to permanently retain these molded geometries in place.^9^^,^^123^ Furthermore, silicone generally remains stable over long periods of time.^121^ Silicone is also flexible, allowing it to emulate the mechanical properties of human skin and other soft tissues.^9^^,^^121^ For example, PDMS can be designed to have a Young’s modulus between 0.1 and 1 MPa, which is in the same range as soft tissue, by altering the concentration of hardeners and plasticizers used.^123^ This can be a beneficial property for contact-based modalities, such as optical fiber probes, and for optical modalities that incorporate or measure mechanical properties, such as photoacoustic imaging or optical coherence elastography, where pressure can alter the measured optical signal.^121^ The mechanical properties and optical properties of silicone can be adjusted by adding absorbing and scattering components and varying the curing temperature or the proportions of base and curing agents.^9^^,^^118^ Specifically, the refractive index of PDMS can be tuned to a range comparable to that of biological tissue by simply varying the curing temperature.^118^ Most silicones are optically transparent across the visible and near-infrared (NIR) range. It is important that any air bubbles that may be present are eliminated prior to curing, allowing for precise tuning of the phantom’s optical properties.

A significant consideration of silicone as a matrix material is its rising cost due to its increasing use in solar panels and other goods.^127^ The popular, high-quality silicone brand SYLGUARD-184 is among the most expensive of the common matrix materials, at typical costs of several hundred US dollars per pound. Recently, there has been a rise in the use of other lower-cost commercially available silicones, such as Smooth-On’s Dragon Skin and PlatSil Siliglass.^73^^,^^128^^,^^129^ Another drawback of silicone is that its inorganic polymer base makes it minimally compatible with organic materials, barring the use of components such as hemoglobin as absorbing agents in these phantoms.^9^^,^^120^

#### 3.1.4 Resin

Another solid matrix material is epoxy resin, which, in its uncured form, is a viscous liquid that can be converted to a hard, durable polymer.^14^^,^^15^^,^^59^^,^^70^^,^^75^^,^^88^^,^^116^^,^^119^^,^^130^^–^^138^ Phantoms made with resin-based materials, generally referred to as resin phantoms, can be made with different resin classes whose polymerization mechanisms influence manufacturing approaches. Thermoset resins are two-part systems consisting of a resin base and a hardener that cure through a chemical reaction upon mixing. This chemical curing process may take hours to days or be accelerated with heat and requires consideration of the working (pot) time—the period after mixing during which the material remains processable for mixing and casting.^15^^,^^116^ Historically, polyester resins have been used in phantom development;^131^^,^^132^^,^^134^ however, in recent years, epoxy resins are more widely used.^15^^,^^59^^,^^70^^,^^116^^,^^133^ These thermoset resin phantoms are fabricated through casting into molds. In contrast, UV-curable resins polymerize via light activation and have gained popularity due to their compatibility with 3D printing, enabling complex geometries and precise control over their design and properties.^75^^,^^130^

Regardless of the polymerization process, resin creates solid, rigid phantoms, allowing for permanently fixed objects with high levels of durability.^138^ Due to their long-term stability and durability, resin-based phantoms are ideal for both routine system calibration and intersystem comparisons.^135^ Furthermore, complex geometries and anatomical features can be achieved with resin through machining, casting epoxy resin around complex molds, and 3D printing UV-curable resin.^138^ With low intrinsic absorption in the visible and NIR spectral regions and weak residual forward-scattering,^137^ resin is highly tunable through the incorporation of other components.^15^^,^^135^^,^^136^ Although resin can mimic the shape of biological structures through advanced manufacturing techniques, it cannot mimic the mechanical properties of soft biological tissue due to its rigidity.^116^ Furthermore, resin cannot emulate the biochemical composition of tissue as it is incompatible with biological additions.^2^ Another optical consideration of resin is that its refractive index, which has been reported at 1.54 to 1.56, is significantly higher than the refractive index of soft biological tissue, which is around 1.35 to 1.4.^136^^,^^137^ Some studies have shown a change in the absorption spectrum of resin over time due to yellowing of the material, which may impact any measurement made in the blue spectral region. This yellowing could affect the effective lifetime of resin-based calibration phantoms, though careful selection of a resin manufacturer should help to prevent or minimize such concerns.^75^^,^^137^^,^^138^

#### 3.1.5 Other common polymers

Several other materials have been utilized as the matrix of optical tissue phantoms. These materials are often selected for specific optical and/or mechanical properties that are not readily achieved with more commonly used materials such as silicone and resin. One example is polyurethane, which creates durable and photostable phantoms with an intrinsic refractive index comparable to that of human skin in the VIS-NIR range.^16^^,^^55^^,^^65^^,^^67^^,^^139^^–^^141^ Polyurethane can be cast and machined, although long curing times are needed for this material.^142^ In addition, polyurethane has stable photoacoustic properties, which mimic those of soft biological tissue.^16^ Other materials including polyvinyl chloride (PVC) and polyvinyl chloride-plastisol (PVC-P) have also been used for tissue-mimicking optical phantoms in oximetry and photoacoustic imaging applications.^51^^,^^124^^,^^142^^–^^146^ These matrix materials have many favorable qualities, such as long-term stability, refractive indices comparable to soft tissue in the VIS-NIR range, and controllable scattering and absorption properties.^142^^,^^145^^,^^146^

### 3.2 Scatterers

In addition to providing its own properties, matrix materials serve as a scaffold for any other materials that are added to modulate the optical properties of the phantom. Typical materials that serve as scattering agents used in optical phantoms fall into three major categories: lipid-based scatterers, metal oxides, and microspheres. Selection of a scattering agent often comes down to a compromise between the wavelength tunability, cost, and the required scattering properties that best match the target tissue. While organic scattering components can more accurately mimic broadband biological spectral properties, increased control over particle size and size distribution can allow for enhanced targeting of scattering behavior at one or more wavelengths of interest. Cost can be an additional factor, especially when using increasingly engineered microparticles. Although cost is not necessarily a constraint for research purposes, it is a significant differentiating factor between these common classes of scatterers, and thus, the tradeoff between cost and precision should be considered, especially when high levels of precision are not required. The relative performance for lipid-based scatterers, metal oxides, and microspheres across these relevant parameters is outlined in Table 2.

**Table 2:** Summary of the three common categories of scatterers used in optical phantoms and their tradeoffs that impact material selection. The categories are relatively ranked for typical cost: least expensive ($) to most expensive ($$$) and favorability in relevant parameters: least favorable (+) to most favorable (+++).

	Cost	Uniform particle size	Availability of different particle sizes	Tissue-mimicking scattering behavior
Microspheres	$$$	+++	+++	+
Metal oxides	$$	++	++	++
Lipid emulsions	$	+	+	+++

#### 3.2.1 Lipid-based scatterers

The most directly biomimicking scattering agent employed in phantoms is lipid-based scatterers, although their optical properties are difficult to control. Lipid emulsions are comprised of small droplets of fat suspended in water. Although milk and nondairy creamers have been employed as lower-cost and widely accessible options, most phantoms utilizing lipid-based scatterers use pharmaceutical sources due to their standardization and consistency between batches.^9^^,^^111^^,^^147^ Commercially available lipid emulsions include Intralipid, Nutralipid, and Liposyn, which are made from soybean oil, egg phospholipids, and glycerol, respectively, with Intralipid being the most common by far.^9^^,^^42^^,^^45^^,^^48^^,^^56^^,^^58^^,^^79^^,^^84^^–^^87^^,^^108^^,^^110^^–^^112^^,^^148^^–^^154^ Intralipid is inexpensive and widely available in 10%, 20%, and 30% fat concentrations. Intralipid 20% is the most commonly used scatterer in tissue optical phantoms and thus has been extensively characterized.^86^^,^^87^^,^^150^ Due to the spherical nature of the fat droplets, the optical properties of lipid emulsions can be predicted by Mie theory.^85^^,^^108^ Consistency in optical properties among different batches of Intralipid 20% is another benefit, with batch-to-batch variations of the intrinsic reduced scattering coefficient reported at only 2%.^87^ This stems from the well-controlled production procedures for Intralipid, ensuring high levels of homogeneity in its properties.^150^ Furthermore, Intralipid is stable for long periods of time, with expiration dates of up to 10 years.^87^^,^^150^ However, there are important considerations in selecting and manufacturing the matrix material used with Intralipid-based phantoms. First, the matrix material must be biocompatible, limiting the options to aqueous suspensions and hydrogels.^151^ Second, the preparation protocol, including mixing time and temperature, has been found to greatly affect the optical properties of Intralipid, necessitating consistent, well-documented, and controlled processes.^151^

#### 3.2.2 Microspheres

Microspheres are the most controlled material that can model the scattering characteristics of tissue in phantoms. Due to their tightly distributed refractive index and size, their scattering properties can be accurately approximated using Mie theory. As such, microspheres serve as highly accurate models for both the scattering coefficient as well as anisotropy of tissue.^107^ Microspheres made of polystyrene are most commonly used as scatterers for tissue phantoms. These are manufactured in size ranges from 110 nm to 10  μm, allowing for careful selection of the wavelength-dependent scattering properties needed.^155^ Silica-based microspheres have also been used as scatterers in tissue phantoms.^108^^,^^115^^,^^135^^,^^136^ Silica microspheres have a methacrylate group at their surface, allowing them to be dispersed in epoxy resin. However, their relatively large size, between 1 and 10  μm, limits their wavelength-dependent scattering properties.^108^^,^^135^^,^^136^ The drawbacks of microspheres as a scattering agent in phantoms include instability of the optical properties of microsphere suspensions over long periods, their high cost in comparison to other scattering agents (typically costing on the scale of hundreds of US dollars for a 5 mL vial), and the high concentrations of the material that may be required to achieve tissue-like scattering properties, ultimately limiting widespread use.^107^^,^^155^

#### 3.2.3 Metal oxides

While microspheres are very controlled in size and shape at the expense of a high cost, metal oxides are a more cost-effective scattering agent. Although metal oxides can approximate Mie scattering to an extent, they do not provide the precision of microspheres.^104^^,^^141^ Regardless, metal oxides are used as a purely scattering agent due to their reproducible, stable, and wavelength-dependent properties in the visible and near-infrared range.^104^^,^^107^^,^^120^^,^^141^ Of the metal oxides readily available, ${\mathrm{TiO}}_{2}$ is the most commonly used metal oxide scatterer due to its high refractive index (∼2.6 at 600 nm), which yields high degrees of scattering at relatively low concentrations.^21^^,^^39^^,^^40^^,^^43^^,^^46^^,^^51^^–^^53^^,^^55^^,^^59^^,^^65^^,^^67^^,^^75^^,^^111^^,^^119^^,^^121^^–^^124^^,^^126^^,^^137^^,^^138^^,^^141^^,^^143^^,^^145^ Other commonly utilized metal oxides include ${\mathrm{Al}}_{2}O_{3}$ and ZnO.^49^^,^^68^^,^^75^^,^^109^^,^^111^^,^^117^^,^^120^^,^^123^^,^^137^^,^^138^^,^^142^^,^^146^ Metal oxides have effective scattering properties due to their high refractive index compared with phantom matrix materials, but the index varies greatly for each metal oxide.^107^ The scattering anisotropy may also vary between the types of metal oxides. Therefore, the selection of the metal oxide will depend on the tissue being mimicked.^59^^,^^134^^,^^141^^,^^156^ For example, the anisotropy of ${\mathrm{TiO}}_{2}$ (g∼0.6) is low compared with the anisotropy of most biological tissues. Furthermore, care must be taken to homogeneously disperse metal oxides in the phantom as they may not easily disperse in certain matrix materials. These scatterers will settle over time and potentially before the phantom has solidified during the curing process, resulting in an inhomogeneous distribution of scatterers.^107^^,^^121^

### 3.3 Absorbers

**Fig. 5:**
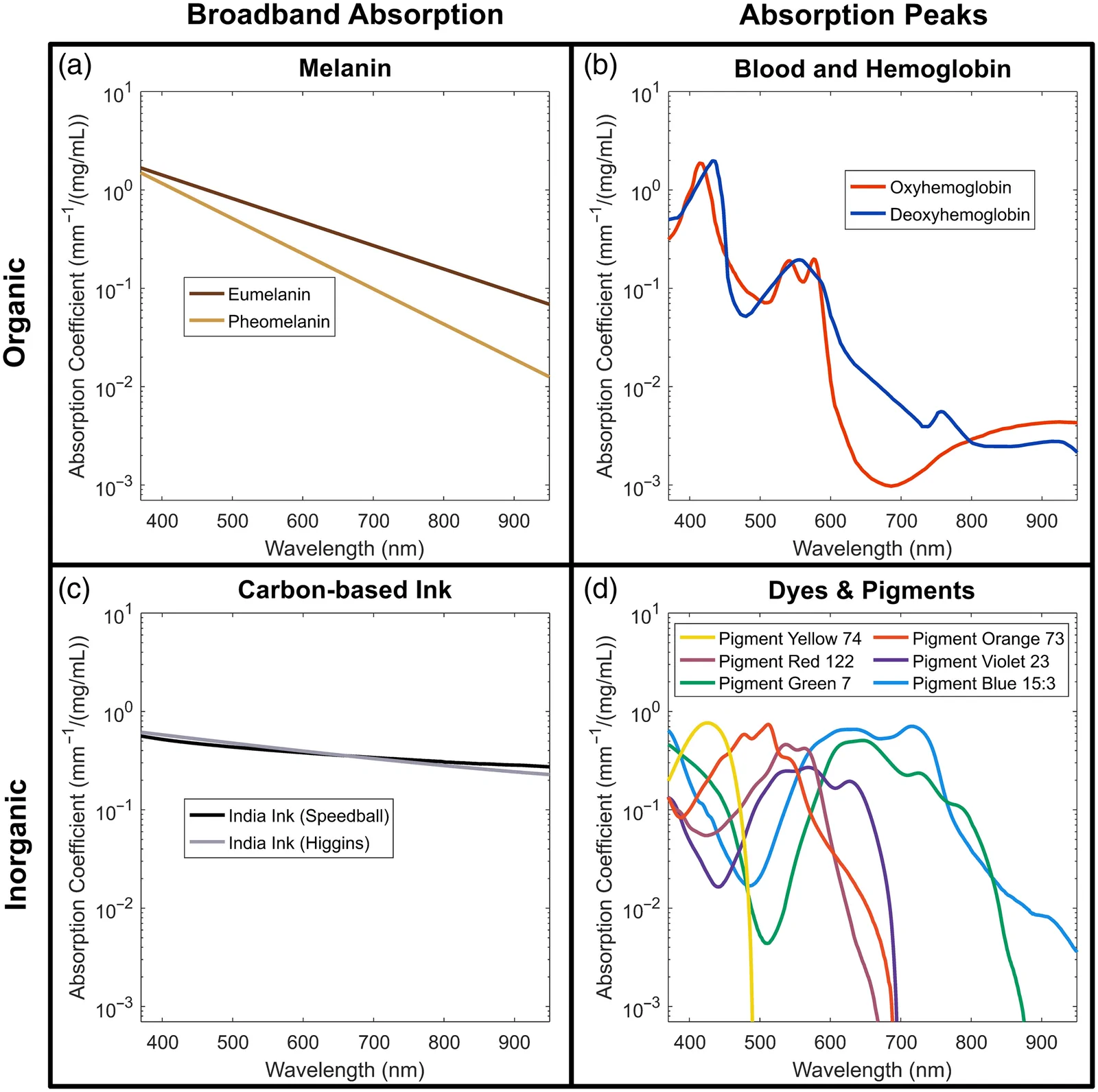
Phantom absorbers in the visible-NIR regions are typically obtained from either organic [(a), (b)] or inorganic [(c), (d)] sources and provide either broadband absorption [(a), (c)] or distinct absorption peaks [(b), (d)]. Although inorganic absorbers often have longer shelf-lives and are easier to incorporate into phantoms, using them to match the wavelength-dependent absorption spectra of tissue is nontrivial. India ink (c) is unable to match the full wavelength dependence of melanin (a), whereas multiple dyes and pigments (d) must be combined together to mimic the shape of hemoglobin (b) absorption. Absorption coefficient spectra were adapted from Refs. 157 and 158.

Beyond scattering, the other major contributor to light attenuation in a material or tissue is absorption. Absorbers are carefully selected to mimic the wavelength-specific absorption coefficients in tissue that are governed by a variety of biological chromophores. These chromophores have both broadband absorption contributions as well as distinct spectral peaks. While water serves as the most common chromophore in the infrared, the main chromophores used for phantom studies in the visible and the NIR are melanin and hemoglobin. Thus, depending on the intended application, phantoms may contain absorbers with broadband absorption, spectral peaks, or both. Although the biological chromophores themselves are often used in tissue optical phantoms, the limit in their use time and incompatibility with many common matrix materials hinder their universal use. Alternative absorbers include a variety of inks, dyes, and pigments, all of which can be stable over long periods of time. The main categories of visible-NIR absorbers used in optical phantoms are summarized in Fig. 5.

#### 3.3.1 Melanin

In the visible and NIR spectral range, melanin is one of the dominant chromophores in human skin and in the retinal pigment epithelium in the eye, and possesses a flat, wavelength-dependent absorption curve, as shown in Fig. 5(a). Melanin is synthesized and stored inside melanosomes within melanocytes. The amount of melanin in melanosomes, the size and distribution of melanosomes, and the type of melanin (eumelanin or pheomelanin) all contribute to variations in skin tone between individuals and across anatomical locations on a single individual.^48^^,^^159^ For optical phantoms that attempt to accurately mimic skin, including melanin directly is the obvious choice. However, complications in isolating, purifying, and handling natural melanin grains, associated costs, and its incompatibility with many matrix materials have limited the widespread implementation of natural melanin in skin phantoms.^117^^,^^145^^,^^160^ Despite these challenges, several studies utilizing sepia melanin, typically isolated from the sacs of cuttlefish, have demonstrated promising results, demonstrating the use of natural melanin as a broadband absorber in tissue mimicking phantoms.^49^^,^^161^^,^^162^

Synthetic melanin has been efficiently implemented in skin phantoms as a more readily available alternative.^40^^,^^46^^,^^163^ However, there are key differences between synthetic and natural melanin that impact their optical properties. Although the addition of melanin is normally driven by a desire to match absorption properties, melanin particles also cause light scattering that must be taken into account. The particle size of the melanin granules used is one factor that determines these scattering effects. Synthetic melanin granules are usually around 30  μm, comprised of clusters of individual spherical melanin granules with 1 to 10  μm diameters, whereas natural melanin is comprised of granules between 0.4 and 0.8  μm.^117^ Furthermore, in human skin, melanin is localized in the basal layer at the dermal–epidermal junction or concentrated in pigmented lesions such as melanocytic neavi (i.e., “moles”). However, when natural or synthetic melanin is incorporated into an engineered phantom layer, it is incorporated throughout the phantom epidermal layer because of the manufacturing limitations in creating a thin layer with melanin.^117^ Furthermore, when mixing melanin with the matrix material, specific protocols need to be followed to prevent clumping of melanin granules and allow even distribution of the absorber, so that uniform absorption properties may be maintained within the epidermal phantom layer.^40^^,^^46^ Despite these issues, synthetic melanin has been used to model the optical contributions of melanin in the skin, including varying skin tone, melanomas, and benign pigmented lesions.^48^^,^^88^^,^^117^^,^^160^^,^^164^

#### 3.3.2 Carbon-based inks

Carbon-based inks, including India ink, carbon black, and graphite, are routinely used to match tissue absorption at a single wavelength. These are often used to mimic tissues that do not have melanin, although they are particularly relevant for absorption in the yellow and red part of the spectrum. Although nigrosine is an organic dye rather than a carbon-based ink, it has similar characteristics and is therefore included here. Carbon-based pigments are sometimes used to represent the flat and broadband absorption of melanin [Fig. 5(c)] as they are widely commercially available and with a cost per gram about 5 orders of magnitude less than synthetic melanin.^108^^,^^135^^,^^165^ While dry inks, alcohol-soluble nigrosine, and graphite are viable broadband absorbers, India ink, carbon black, and water-soluble nigrosine are the most commonly used absorbers that produce stable, broadband, and homogeneous optical properties.^3^^,^^9^^,^^10^^,^^16^^,^^39^^,^^45^^,^^46^^,^^50^^,^^52^^,^^53^^,^^55^^,^^65^^,^^67^^,^^75^^,^^79^^,^^85^^,^^87^^,^^88^^,^^107^^–^^110^^,^^112^^,^^119^^,^^121^^–^^124^^,^^126^^,^^145^^,^^146^^,^^148^^,^^149^^,^^155^^,^^165^^,^^166^ Optical properties of these inks are consistent, reproducible, and stable over long periods of time.^165^

However, when using India ink or other commercially available carbon-based inks, one must be aware of brand-to-brand and batch-to-batch variations in optical properties.^108^^,^^165^ India ink from many manufacturers has been well characterized in Di Ninni et al., resulting in the conclusion that although the specific values for the absorption coefficient and extinction coefficient, which accounts for overall light attenuation and incorporates both absorption and scattering, differ across ink brands, the ratio of these two values remains consistent. The consistency across the relative optical properties of different inks is also seen in the reported single-scattering albedos, which is a ratiometric property of scattering to the overall extinction coefficient in a single scattering event.^165^ However, when working with India ink, the ink and other nonchemically pure additives should be fully characterized before use, with the target absorption then being obtained through dilution.^108^

#### 3.3.3 Blood and hemoglobin

Hemoglobin is a dominant absorber in tissue across the visible and NIR ranges, with characteristic wavelength-dependent peaks near ∼414, ∼542, and ∼576  nm for oxyhemoglobin (${\mathrm{HbO}}_{2}$) and ∼430 and ∼556  nm for deoxyhemoglobin (Hb), as shown in Fig. 5(b).^158^ Whole blood absorption arises primarily from hemoglobin and water. As oxygen binding alters hemoglobin’s optical characteristics [Fig. 5(b)], the hemoglobin oxygenation state in the target tissue must be considered in phantom design. In phantoms modeling *ex vivo* tissues or surgical fields where electrocautery may be present, deoxyhemoglobin should be explicitly considered.^158^^,^^167^ Blood and hemoglobin are commonly used in phantoms to reproduce full-spectrum tissue absorption.^17^^,^^39^^,^^45^^,^^48^^,^^56^^,^^58^^,^^81^^,^^84^^,^^85^^,^^87^^,^^91^^,^^105^^,^^109^^,^^156^^,^^163^^,^^164^ However, as with melanin, incorporating biological absorbers requires attention to multiple factors to ensure consistent and accurate optical properties.

The source and form of blood used may influence the phantom optical properties.^86^ Whole blood—drawn directly from animals or human participants—can be used in phantoms and provides broadband absorption with some scattering from intact erythrocytes.^83^^,^^86^^,^^168^^,^^169^ In this case, anticoagulants (e.g., heparin, CPDA) are required to prevent clotting. Methemoglobin can form as samples age, dampening the expected spectral peaks and impacting phantom accuracy.^86^^,^^170^ Methemoglobin can be chemically reduced back to deoxygenated, partially oxygenated, or fully oxygenated states using sodium dithionite, or its presence can be accounted for in models of light attenuation used to analyze the measurements.^86^^,^^171^ Biosafety training and equipment are necessary when handling whole blood.^86^ Lysed blood is also used in tissue phantoms and minimizes light scattering by breaking down cells, yielding a primarily absorbing material.^86^ Freeze-dried bovine and human hemoglobin are common commercial alternatives that reduce biosafety risks and complexity in handling.^58^^,^^170^^,^^171^ However, freeze-drying frequently introduces methemoglobin that can dominate the sample, with levels varying by manufacturer, storage, and age.^86^^,^^170^^,^^171^

Incorporating blood often necessitates biocompatible matrix materials, typically liquid (Sec. 3.1.1) or gelatin (Sec. 3.1.2), which trade stability for biocompatibility. These organic matrices generally have limited short-term stability. Progress has been reported with hydrogel phantoms containing various forms of blood.^58^^,^^89^^,^^171^ Sodium azide has been used as a preservative in blood-containing gelatin phantoms to stabilize NIR optical properties for up to four weeks.^58^

Liquid phantoms are particularly suited to mimic living tissue because oxygen saturation—and thus absorption—can be controlled and adjusted over time.^83^^,^^84^^,^^86^^,^^168^^–^^170^^,^^172^ The oxygen saturation in the phantom (ratio of oxyhemoglobin to total hemoglobin concentration) may need to match the target tissue. Dynamic control is useful for perfusion studies and characterization of oxygenation measurement devices.^56^^,^^58^^,^^83^^,^^86^ Two common deoxygenation methods are yeast addition and $N_{2}$ bubbling, with partial pressure of oxygen (${\mathrm{pO}}_{2}$) measurements used to validate the oxygenation state of the phantom. Yeast consumes oxygen to produce deoxyhemoglobin, enabling controlled deoxygenation that can be monitored optically.^83^^,^^84^^,^^89^^,^^172^^,^^173^ For liquid phantoms, yeast-based deoxygenation requires close control of pH and temperature at physiological values (pH of 7.4 and 37°C).^83^ Gelatin phantoms have also employed yeast for deoxygenation.^89^ In liquid phantoms, glucose can be added to further enhance oxygen consumption.^168^ Alternatively, bubbling $N_{2}$ gas into the phantom washes out the dissolved oxygen.^168^^,^^169^ Complete deoxygenation by $N_{2}$ typically requires incremental flow of $N_{2}$ and longer times than yeast.^168^ This method requires an air-tight phantom container, and the gas often cools the phantom, may cool the phantom (complicating physiological temperature control), and can alter the properties of some scatterers due to mechanical stress on liquid droplets.^168^ Re-oxygenation can be achieved by bubbling $O_{2}$ regardless of the deoxygenation method.^84^

Phosphate-buffered solution (PBS) is a common base liquid for blood-containing phantoms as it maintains pH stability and matches physiological osmolarity and ion concentrations.^84^^,^^168^^,^^169^^,^^172^ Isotonic saline is also used as a biocompatible matrix for erythrocytes to achieve accurate absorption behavior.^174^ However, its chemical differences from plasma make it suboptimal for chemically specific optical sensing techniques in the NIR (e.g., fluorescence imaging or Raman spectroscopy).^174^ Despite their primary function in phantom design as scattering additions, lipid emulsions also commonly serve as the matrix material for blood phantoms.^83^^,^^84^^,^^168^^,^^169^^,^^172^

For long-term stability and standardization applications, integrating blood constituents into silicone matrices (Sec. 3.1.3) is desirable but challenging due to silicone hydrophobicity and high curing temperatures.^86^ Several strategies have been explored, generally yielding fixed absorbance and oxygenation after curing.^86^ One approach uses freeze-dried hemoglobin as a pigment, with vigorous mixing and extended sonication in PDMS base to disperse submicron particles while preserving hemoglobin’s chemical structure.^170^ Another approach sonicates purified hemoglobin solutions into silicone, followed by curing at low temperatures to avoid denaturation; this has been demonstrated with both oxyhemoglobin and deoxyhemoglobin, maintaining stable spectral peaks for over four months.^173^ A third approach encapsulates purified hemoglobin within microcapsules fabricated via liquid-driven coaxial flow focusing and embeds them in silicone.^175^ The photocurable shell yields microcapsules with narrow size distributions and permeability to water and gases, enabling control of oxygen binding while protecting the hemoglobin core.^175^ These microcapsule phantoms exhibit visible-range stability for at least 25 weeks and allow modeling of deoxygenation dynamics due to oxygen permeability through the thin resin shell.^175^

#### 3.3.4 Dyes and pigments

Some tissue phantoms need to be inorganic for their intended use or due to restrictions of compatibility with the matrix material selected while still requiring absorption peaks at specific wavelengths or regions of the visible and NIR spectrum. In these cases, it is common to use dyes and pigments, either as pure components or as composites with other materials in the form of inks and paints, as absorbing components in phantoms.^15^^,^^42^^,^^51^^,^^59^^,^^88^^,^^125^^,^^137^^,^^138^^,^^141^^,^^176^ While composite materials, such as paints, pastes, and colored inks, often contain stabilizers, emulsifiers, and other components that can improve the incorporation of both dyes and pigments in phantoms compared with their pure forms, there are also key differences between dyes and pigments that should be considered in phantom design.

Pigments are fine powders that can be dispersed in a medium to affect the color through distinct absorption and/or scattering properties.^177^ Specifically, the range of colors and chemical compositions in pigments allows for the selection of specific absorption peaks and spectral shapes.^15^^,^^177^ Strictly speaking, metal oxides and carbon-based inks are also pigments: white pigments with broadband scattering behavior and black pigments with broadband absorption, but due to their unique role in tissue optical phantoms, these are discussed elsewhere in this paper. Pigments are insoluble and thus remain as intact particulates in the phantom matrix, giving them nonnegligible scattering properties that can be expected to vary between different pigments and different commercial sources.^177^ Therefore, the scattering properties of pigments should also be characterized when used as an absorbing agent to ensure precise phantom design.^15^^,^^137^^,^^177^ Pigments are generally lightfast, meaning their optical properties stay unaffected in a phantom even with extensive use, with some pigmented phantoms maintaining stable optical properties over periods of up to 12 months.^125^ Most commercial pigments have visible absorption as they are made to provide color, though many materials have been found to have near- and short-wave-infrared absorption as well, broadening their use.^137^ Pigments have been used to simulate the optical properties of skin and soft tissues, with groups often combining several pigments with distinct absorption characteristics to achieve the desired spectral behavior, as shown in Fig. 5(c).^15^^,^^125^^,^^137^

In contrast, dyes are soluble in their base material, whether that is oil, organic solvents, or water, and therefore, disperse in the matrix material at a molecular level, ultimately contributing to the absorption of light by the phantom.^88^^,^^135^^,^^136^^,^^138^^,^^141^^,^^155^^,^^177^ Due to their solubility, properly utilized dyes tend to provide minimal to no scattering effects in phantom models. Combinations of dyes with narrow absorption bands have been used to model the spectral characteristics of chromophores such as hemoglobin or blood, although dyes with overlapping absorption bands may prevent the desired optical behavior.^138^^,^^141^ The incorporation of dyes in an optical phantom may place restrictions on the matrix material selection. For example, some dyes may fade or photobleach after periods of light exposure or from the heat caused by the curing process of resins and other plastic-based polymers, ultimately impacting the stability and optical properties of the phantom.^136^^,^^138^^,^^141^ Depending on the type of matrix material and dyes, color may diffuse between layers of multilayered phantoms or phantoms with inclusions, causing a shift in the desired optical properties.^141^ However, selecting photostable dyes and compatible matrix materials can mitigate these concerns and allow stable optical phantoms.

#### 3.3.5 Water

In addition to the previously discussed pigments, biological chromophores, and dyes, we would be remiss not to include water as an important chromophore. In most biomedical optical systems, wavelengths in the visible or NIR parts of the spectrum are utilized to target specific biological chromophores or increase the probing depth of the system by making use of the “water window,” the area of the optical spectrum from ∼600 to 900 nm where water absorption is relatively low. However, there are a number of optical systems and applications that target the absorption of light by water as their primary contrast or interaction mechanism. Water has a number of strong absorption peaks that, depending on the wavelength used, provide absorption coefficients ranging over 5 orders of magnitude.^178^ Important absorption peaks include 980 nm ($\mu_{a}=0.45\;{\mathrm{cm}}^{-1}$); 1440 nm ($\mu_{a}=28.8\;{\mathrm{cm}}^{-1}$); 1.94  μm ($\mu_{a}=119.8\;{\mathrm{cm}}^{-1}$); and 2.94  μm ($\mu_{a}=12,691\;{\mathrm{cm}}^{-1}$).

Water-based tissue phantoms are used for ablation studies in which strongly absorbed laser wavelengths are used or that are based on short-pulse, laser-induced breakdown mechanisms.^113^^,^^179^^–^^181^ While these phantoms may be strongly absorbing in the infrared part of the spectrum, they are typically transparent in the visible, allowing detailed visualization of the spatio-temporal evolution of the ablation process, including pressure transients, shockwaves, explosive events, and collateral damage. In these applications, water acts both as the absorber and the matrix material, and the thermal and mechanical properties of the phantom need to be carefully considered in addition to the optical properties. By varying the water concentration and by adding structural components, such as polyacrylamide, collagen fibers, or other scaffold materials, the researcher can control the mechanical properties of these phantoms. Similarly, by replacing water with deuterated water ($D_{2}O$), one can control the optical IR absorption while keeping the thermal and mechanical properties constant.

Aside from making elegant ablation phantoms, water can also be used as an important chromophore in phantoms used in short-wave infrared (SWIR) spectroscopic studies that typically operate in the 0.9 to 2.0  μm wavelength range.^182^ This part of the optical spectrum has unique spectral signatures that allow characterization of a variety of important biological molecules, such as lipids, collagen, glucose, and water itself, while benefiting from the relatively low tissue scattering at these wavelengths. In addition, although not acting as an absorber, water is still an important contrast component in phantoms for Raman spectroscopy. This is particularly true for high-wavenumber Raman spectroscopy (2800 to $3800\;{\mathrm{cm}}^{-1}$), which contains information on oxygen–hydrogen stretching modes and is highly sensitive to tissue water content. As with other optical modalities, tissue optical phantoms are often used and needed for the calibration and validation of the accuracy and sensitivity of these systems. The use of well-controlled gelatin hydrogel phantoms has been shown to provide physiologically relevant contrast with fine enough control of the composition for quantitative performance assessments.^40^^,^^41^^,^^92^

## 4 Modalities

Different modalities have specific needs for the structural, mechanical, optical, and biochemical properties, depending on the method of light–matter interaction for its mechanism of action. The general categories of the phantom geometries and material choices and their tradeoffs have been previously described in Secs. 2 and 3, respectively. Here, we will present optical property considerations, material requirements, and geometry considerations in designing phantoms for various optical modalities as a representation of the process to be considered in designing modality-specific phantoms. Although it is not feasible to address all optical modalities, this section will cover design considerations for five modalities, including diffuse optics, fluorescence imaging, photoacoustic imaging, optical coherence tomography, and Raman spectroscopy. Table 1 should be referenced for more specific reviews on phantoms for the modalities we discuss here and more modalities beyond these examples. In addition to describing modality-specific considerations, we provide a snapshot of recently developed phantoms, including their designs and material choices, for each of these modalities in Tables 3–7.

### 4.1 Diffuse Optical Phantoms

The most commonly used phantoms in the field of biophotonics are those developed for diffuse reflectance and other optical modalities that utilize light diffusion for their measurements. In these approaches, light at the wavelength(s) of the incident light source is modulated by the absorption and scattering properties of the medium and is collected in reflectance or transmission mode. Thus, these phantoms depend primarily on their optical properties at the wavelength(s) of the incident light and are therefore relatively straightforward in their design. Such phantoms are widely used to calibrate signal collection, validate measurement accuracy, and perform a wide range of system characterizations, including resolution, linearity, stability, contrast, noise, responsivity, and sensitivity.^15^^,^^18^^,^^42^^,^^48^^,^^49^^,^^83^^,^^84^^,^^117^^,^^153^^,^^199^^,^^200^ Furthermore, three major protocols have been developed that use one or more phantoms to perform performance assessments and standardizations of diffuse optical instruments:

1) The basic instrumental performance (BIP) protocol focuses on characterizing the response function and responsivity of an instrument or system using a solid, homogeneous diffuse transmission phantom.^201^
2) In contrast, the MEDPHOT protocol utilizes a set of 32 different phantoms with optical properties spanning four levels of scattering, from 0.5 to $2.0\;{\mathrm{mm}}^{-1}$ at 800 nm, and eight levels of absorption, from 0 to $0.035\;{\mathrm{mm}}^{-1}$ at 800 nm, to characterize the ability of diffuse optical instruments to recover optical properties, thereby assessing their accuracy, linearity, stability, and reproducibility.^199^
3) Last, the nEUROPt protocol characterizes the ability of an instrument to detect, localize, and quantify optical inhomogeneities in diffuse media using either a liquid phantom with suspended, absorbing inclusions or a solid phantom with a mechanically replaceable inclusion.^200^^,^^202^

A combination of these protocols has been shown to provide a robust analysis of the hardware performance across a wide range of diffuse optical instruments. However, refinement and validation will be needed before a consensus is reached for this field.^203^

#### 4.1.1 Optical property considerations

While diffuse optical phantoms tuned to a single wavelength are common and sufficient for many applications, multiwavelength dependence may be desirable or even required for certain applications.^15^^,^^42^^,^^84^^,^^112^^,^^199^ Spectral matching is required for hyperspectral imaging, color matching, and reflectance spectroscopy, including most optical devices used in oximetry.^15^^,^^16^^,^^18^^,^^48^^,^^49^^,^^83^^,^^117^^,^^138^^,^^188^ Generally, it is difficult to achieve tissue-like spectral properties without using biological chromophores.^18^^,^^48^^,^^49^^,^^83^^,^^84^ This has led to a push, in recent years, toward combining multiple dyes or pigments to provide shelf-stable spectral characteristics and spectral matching, particularly in the visible and NIR regions [Figs. 5(c) and 5(d)].^15^^,^^16^^,^^117^^,^^138^ The most realistic broadband phantom (390 to 950 nm) to date was generated using weighted linear combinations of up to 20 different pigments dispersed in a solid matrix [Fig. 2(c)].^15^

#### 4.1.2 Material requirements

Phantoms for diffuse optics applications can be composed of liquid or solid and organic or synthetic matrix materials, or a combination of materials, such as a liquid with solid inclusions. The optimal bulk matrix depends on the use case for the phantom and the requirements of the desired measurement outcomes. Liquid phantoms (Sec. 3.1.1) for diffuse optics are typically aqueous emulsions used mainly for single-use experiments in which a large range of properties is required.^42^^,^^83^^,^^84^^,^^153^^,^^200^ A common example of liquid optical phantoms includes phantoms used to test oximetry devices, as the oxygenation level can be controlled, as described in Sec. 3.3.3. Solid phantoms may be desired to mimic the physical properties of tissue, such as layers or inclusions with varying optical characteristics, or if the phantom is to be used multiple times. These phantoms are made with an organic hydrogel matrix (Sec. 3.1.2), with the addition of biological chromophores as required.^18^^,^^48^^,^^112^ Silicones, resins, and plastic-based matrix materials are used for diffuse optics phantoms when long-term stability is desired, and are described in depth in Secs. 3.1.3–3.1.5.^15^^,^^16^^,^^49^^,^^117^^,^^138^^,^^199^^,^^201^ While these general guidelines for material choice apply across diffuse optics phantoms, additional requirements need to be considered for specific techniques. For example, shortwave infrared (SWIR) phantoms are limited to using water and lipids as their primary absorbers due to the dominance of their absorption contributions in tissue at that wavelength range.^18^ However, the SWIR absorption characteristics of dyes and pigments (Sec. 3.3.4) have recently been characterized, potentially opening up the viability of more inorganic SWIR phantoms.^18^^,^^137^^,^^204^

#### 4.1.3 Geometry considerations

Phantoms are typically homogeneous when used for system calibration and characterization of diffuse optics instruments.^15^^,^^18^^,^^42^^,^^83^^,^^112^^,^^138^^,^^153^^,^^199^^,^^201^ More intricate phantoms are used with multiple layers and complex geometries to mimic specific anatomical structures and biological phenomena.^16^^,^^18^^,^^48^^,^^49^^,^^84^^,^^117^^,^^200^ Multilayered phantoms are required when simulating the optical effects of skin tone due to the localization of chromophores, namely, melanin and blood, in the epidermis and dermis, respectively, as will be discussed in more detail in Sec. 6.^16^^,^^48^^,^^49^^,^^117^

**Table 3:** Selected publications focusing on the development of optical phantoms for diffuse modalities over the previous 5 years (2020 to 2025).

Authors	Year	Matrix material	Absorber(s)	Scatterer(s)	Geometry	Other	Reference
Barbosa da Cruz et al.	2025	Epoxy resin	Inorganic pigments	${\mathrm{TiO}}_{2}$ and powdered minerals	Homogeneous	Commercial cosmetic powder use to mimic skin tones	183
Bhusal et al.	2025	Silicone	India ink	${\mathrm{TiO}}_{2}$	Anatomical with inclusions	Matched tissue mechanical properties	184
Chatterjee et al.	2025	Silicone and agarose hydrogel	—	—	Anatomical	Generative design of complex vasculature	185
Dreher et al.	2025	Copolymer-in-oil	Inorganic pigments and dyes	${\mathrm{TiO}}_{2}$	Anatomical	Combination of absorbers to match different blood oxygenation states	186
Ragunathan et al.	2025	Thermoplastic	—	—	Homogeneous, patterned, and anatomical	Thermoplastic 3D printing using a filament-mixing extruder	69
Jayasankar et al.	2024	Silicone	FAD and NADH	Glycerol	Patterned and layered	—	187
Kim et al.	2024	Agar hydrogel	Nigrosin	Intralipid	Homogeneous	—	110
Ma et al.	2024	Silicone	Carbon black	Silica microspheres	Layered	Dynamic modulation using an integrated LCD	188
Rodriguez et al.	2024	Silicone and UV resin	Nigrosin	${\mathrm{TiO}}_{2}$	Phantoms with inclusions	—	124
Jenne et al.	2023	Silicone	Synthetic melanin, India ink	${\mathrm{TiO}}_{2}$	Layered with inclusions	Phantom integrated with artificial circulatory system	46
Lin et al.	2023	Epoxy resin	Carbon black	${\mathrm{TiO}}_{2}$	Layered	Dynamic modulation using an integrated LCD	189
Morsink et al.	2023	Polyurethane	Inorganic pigments	—	Homogeneous	Made using an office laser printer	16
Walter et al.	2023	Epoxy resin	Inorganic pigments, India ink	${\mathrm{TiO}}_{2}$, ZnO, and ${\mathrm{Al}}_{2}O_{3}$	Homogeneous	Combination of up to 20 pigments to match wavelength-dependent tissue optical properties	15
Zhao et al.	2023	Silicone	Inorganic pigments	Metal oxides	Anatomical with inclusions	—	125
Afshari et al.	2022	Silicone and UV resin	Blood and nigrosin	${\mathrm{TiO}}_{2}$	Layered with inclusions	Deoxygenation obtained using sodium dithionite	162
Amiri et al.	2022	Gelatin, agar, and polyvinyl chloride hydrogels	Water, lard	Lard	Homogeneous and anatomical	—	18
Waks Serra et al.	2022	Silicone	Carbon black	${\mathrm{TiO}}_{2}$	Anatomical with inclusions	—	190
Goldfain et al.	2021	Silicone	Carbon black	${\mathrm{TiO}}_{2}$ and polystyrene microspheres	Homogeneous	—	123
Majedy et al.	2021	Water	Blood and hemoglobin	Intralipid	Homogeneous	Deoxygenation obtained using live yeast	86
Zhao et al.	2021	Epoxy resin	Inorganic dyes	${\mathrm{TiO}}_{2}$	Anatomical with inclusions	Dynamic modulation using a thermochromic dye	191
Naglič et al.	2020	Silicone	Carbon black	Silica microspheres	Homogeneous	Investigated sub-diffuse scattering properties	129
Ntombela et al.	2020	Agar hydrogel	India ink	${\mathrm{Al}}_{2}O_{3}$	Homogeneous	—	109
Pacheco et al.	2020	Silicone and UV resin	India ink	Silica microspheres	Anatomical	Reconstruction of a neonatal thorax	192

### 4.2 Fluorescence Imaging

Early iterations of fluorescence instruments, such as the spectrofluorometer, used varying concentrations of rhodamine dye (Rhodamine B or 6G) in ethylene glycol as a reference standard. With the growth of fluorescence in biophotonics research, more complex fluorescence phantoms have been developed to characterize spectroscopic and imaging systems, as well as to perform system comparisons between academic and commercial instruments.^13^^,^^19^^,^^63^^–^^66^ Depending on the specific phantom, a wide range of performance metrics can be determined, including illumination homogeneity, flat field corrections, spectral and spatial resolution, field of view, depth of field, signal stability, signal linearity, and limit of detection.^13^^,^^14^^,^^50^^,^^63^^,^^66^^,^^67^^,^^154^ Furthermore, how the surrounding optical properties and the depth of the fluorescence signal affect the overall intensity, the signal-to-background ratio, and the signal-to-noise ratios can also be elucidated.^13^^,^^14^^,^^19^^,^^50^^,^^56^^,^^57^^,^^63^^,^^66^^,^^67^^,^^154^^,^^163^ Fluorescent phantoms have also been used as a calibration model to predict fluorophore concentration in tissue.^68^^,^^163^ While fluorescence phantoms should have optical properties similar to those of the tissue of interest, they must additionally yield fluorescence that is similar in intensity and emission wavelength to the fluorophore of interest. This imposes a number of constraints on phantom design, in both the material and geometric design, that must be addressed before phantom fabrication.

#### 4.2.1 Optical property considerations

When designing a phantom for fluorescence, it is important to consider the optical properties of the phantom at both the excitation wavelength and emission wavelength(s).^59^^,^^163^ However, designing broadband fluorescence phantoms with accurate properties at multiple wavelengths requires careful selection of phantom materials and must undergo extensive characterization processes before they can be implemented.^75^^,^^149^ A simpler approach is to design phantoms where either the excitation or emission wavelength is targeted, though this only provides partial information about the system. Whether the target wavelength for the phantom is the excitation or emission wavelength varies between groups and use cases.^13^^,^^19^^,^^50^^,^^56^^–^^58^^,^^67^^,^^154^ Another consideration in designing fluorescence phantoms is the effects that a high concentration of fluorophores can have on the optical properties of the phantom as a whole. When low concentrations of fluorophore are used, it can be assumed that their inclusion in a phantom with tissue-like optical properties minimally alters the optical properties. However, when concentrations are high, fluorophore absorption can be expected to significantly alter the effective absorption coefficient of the phantom.^205^ This issue is particularly significant when working with autofluorescence phantoms, where wavelength selectivity for both excitation as well as emission becomes important.^57^^,^^66^^,^^68^^,^^149^^,^^163^

#### 4.2.2 Material requirements

A wide variety of matrix materials, including liquid, solid, organic, and synthetic, are used for fluorescence phantoms, with the primary constraining factors being the compatibility of the matrix with the fluorophore of interest, potential autofluorescence from the matrix material itself, and the desired application. The target fluorophores in phantoms are often exogenous agents, though, in some situations, autofluorescence from collagen, NADH, tryptophan, or FAD may be used.^68^^,^^149^^,^^163^ As gelatin exhibits broad autofluorescence, interactions between the exogenous fluorophore and this matrix material during the fabrication process may result in a change in the resulting phantom fluorescence, which should be considered in addition to other factors discussed in Sec. 3.1.2.^75^ Liquid phantoms (Sec. 3.1.1) are routinely used when uniform fluorescence is required or for blood kinetics studies, though the specific liquid solvent used must be compatible with the fluorophore of interest.^14^^,^^57^ Solid phantoms are typically organic hydrogels (Sec. 3.1.2) when the target fluorophore is biologically relevant.^50^^,^^56^^,^^58^^,^^68^^,^^149^^,^^154^^,^^163^ Inorganic matrix materials, commonly PDMS (Sec. 3.1.3) and resin (Sec. 3.1.4), have been used to improve shelf-life and enable complex geometries, although they limit compatibility with biological fluorophores.^13^^,^^19^^,^^59^^,^^66^^,^^67^^,^^75^ This, coupled with the challenge of photobleaching due to the relatively low photostability of most biological fluorophores, results in some phantoms utilizing a different fluorophore as a more stable representative than what would be used clinically. These photostable fluorophores are often either quantum dots or engineered fluorescent dyes that enable the phantoms to achieve a level of material compatibility, shelf-life, and affordability that cannot be matched using the biological fluorophore. Given that the representative fluorophore has similar excitation and emission properties, phantoms made with these materials are highly effective for longitudinal and intersystem calibration and characterization.^13^^,^^19^^,^^50^^,^^58^^,^^66^^,^^67^^,^^75^^,^^149^^,^^154^

#### 4.2.3 Geometry considerations

Depending on the desired use case, fluorescence phantoms range from homogeneous to highly structured. Homogeneous phantoms are useful for basic system calibration, including performing source compensation, analyzing signal strength and linearity, as well as performing flat field corrections, stability measurements, and light leakage tests.^66^^,^^68^^,^^206^ In recent years, there has been a push to develop patterned phantoms that provide all required metrics, either in a stand-alone, composite phantom or in a small set of phantoms [Fig. 2(a)]. Composite fluorescent phantoms have been developed for calibrating concentration dependence, optical property dependence, depth dependence, resolution, illumination homogeneity, and light leakage.^13^^,^^63^^–^^65^^,^^67^ In contrast, more complex, structured phantoms, including multilayered models and anatomically accurate geometries, provide improved depth-dependent analogs for tissue fluorescence resulting in novel metrics of characterization.^13^^,^^14^^,^^19^^,^^67^^,^^206^ For example, thin layers of material surrounding fluorescent inclusions are often used to characterize how the measured fluorescence signal varies with distribution and depth of the fluorophore, such as in skin.^50^^,^^56^^–^^59^^,^^149^^,^^154^^,^^163^ Molding and 3D-printing allow anatomical accuracy in fluorescent inclusions, such as flow channels embedded in phantom matrixes for blood-based and perfusion fluorophores [Fig. 2(b)] or fluorescing geometrical shapes (e.g., spheres and ellipsoids) to represent fluorescently labeled tumors or lymph nodes.^14^^,^^19^^,^^56^^,^^57^^,^^75^ Such inclusions can be incorporated at various depths to mimic the spatial distribution of fluorophores in the biological target of interest.^13^^,^^14^^,^^50^^,^^57^

**Table 4:** Selected publications focusing on the development of optical phantoms for fluorescence modalities over the previous 5 years (2020 to 2025).

Authors	Year	Matrix material	Absorber(s)	Scatterer(s)	Geometry	Other	Reference
Chavez et al.	2025	Silicone and agar hydrogel	—	${\mathrm{TiO}}_{2}$	Phantom with inclusions	—	207
Harun et al.	2025	Gelatin hydrogel	Hemoglobin	Intralipid	Anatomical	Mimics tumor mechanical properties and microenvironment	208
Lee et al.	2025	Silicone	Inorganic pigment	${\mathrm{TiO}}_{2}$ and intralipid	Anatomical	—	209
Mannoh et al.	2025	UV Resin	—	—	Patterned	—	210
Yang et al.	2025	Glass	Silver nanoparticles	Air	Patterned and layered	—	211
Ghauri et al.	2024	Silicone, water, and gelatin hydrogel	Carbon black and India ink	${\mathrm{TiO}}_{2}$ and intralipid	Anatomical	Different phantom organs realized using different matrix materials	212
Jayasankar et al.	2024	Silicone	FAD and NADH	Glycerol	Patterned and layered	Mimics tissue autofluorescence	187
Gibson et al.	2023	Gelatin hydrogel	Hemoglobin and inorganic dyes	Intralipid	Homogeneous and patterned	Combines tissue autofluorescence and exogenous fluorescence	154
LaRochelle et al.	2023	UV resin	Hemin and nigrosin	${\mathrm{TiO}}_{2}$	Anatomical with inclusions	ICG-equivalent fluorophore	19
Freymüller et al.	2022	Water	—	Polystyrene spheres	Patterned	Fluorescent microspheres	213
Khilov et al.	2022	Agar hydrogel	Inorganic pigment	Intralipid	Layered	—	214
Schädel-Ebner et al.	2022	UV resin	India ink	${\mathrm{TiO}}_{2}$	Anatomical and homogeneous	ICG-equivalent fluorophore	215
Horng et al.	2021	Thermoplastic	—	—	Patterned and anatomical	Direct laser writing of small-scale channels	216
Ruiz et al.	2021	UV resin	Hemin and nigrosin	${\mathrm{TiO}}_{2}$	Patterned and anatomical	ICG-equivalent fluorophore	75
Shupletsov et al.	2021	Gelatin and polyacrylamide hydrogel	—	ZnO	Homogeneous	Additional florescence provided by FAD	68
Gorpas et al.	2020	Polyurethane	Hemin and nigrosin	${\mathrm{TiO}}_{2}$	Patterned	Quantum dot fluorescence	64
Kanniyappan et al.	2020	Epoxy resin and UV resin	—	${\mathrm{TiO}}_{2}$	Homogeneous and patterned with inclusions	—	217
Lu et al.	2020	Water	India ink	Milk	Homogeneous	—	82
Ruiz et al.	2020	Polyurethane	Hemin	${\mathrm{TiO}}_{2}$	Patterned	ICG-equivalent fluorophore	13

### 4.3 Photoacoustic Imaging

Phantoms for photoacoustic imaging (PAI) have a wide range of applications, including verifying device specifications, validating accuracy and precision drift over time, quantitatively comparing devices, and as a long-term reference standard.^16^^,^^22^^,^^51^^–^^53^^,^^60^^,^^143^^,^^145^^,^^218^ In addition, there has been a strong push in the PAI community to come to a consensus on acceptable standards required for tissue-mimicking phantoms in the field. The development of the International Photoacoustic Standardization Consortium (IPASC) has centralized recommendations for the development of PAI phantoms. These recommendations, particularly focused on material recommendations, often set requirements for mechanical, acoustic, and thermal properties, in addition to the optical properties.^30^ Material requirements for the phantom matrix and inclusions are vital for the success of PAI as realistic contrast is necessary.

#### 4.3.1 Optical property considerations

As PAI can be performed using a wide range of wavelengths from the visible to the NIR, with multispectral approaches enabling chromophore identification, phantoms should ideally have spectrally matched properties.^20^^,^^61^^,^^143^^,^^160^ In practice, however, optical properties are generally set to only match at one specific wavelength.^16^^,^^51^^–^^53^^,^^218^ In many cases, the isosbestic point of hemoglobin, 805 nm, is commonly used as a reference wavelength in PAI phantoms to relate acquired signals to expected chromophore concentrations.^53^^,^^145^^,^^160^^,^^218^

#### 4.3.2 Material requirements

Due to the role of both optical and acoustic properties in PAI, matrix materials for PAI phantoms typically allow for embedded inclusions to serve as imaging targets. For high tunability and accurate matching of optical and acoustic properties, hydrogels (Sec. 3.1.2) have been used as matrix materials.^52^^,^^53^^,^^160^ For longer-term stability, both polyurethane and PVCP (Sec. 3.1.5) have historically been used, though many of the newer phantom designs have opted to use copolymer-in-oil, a recently described matrix material.^16^^,^^20^^,^^22^^,^^39^^,^^51^^,^^60^^,^^61^^,^^143^^,^^145^^,^^218^^–^^220^ Comprised primarily of mineral oil with added stabilizing and solidifying polymers, copolymer-in-oil, or gel wax, phantoms can be tuned to match both optical and acoustic properties of tissue while remaining stable over long periods of time.^20^^,^^39^^,^^61^^,^^72^^,^^218^^,^^221^ To match optical properties of tissue, the scattering behavior of PAI phantoms is often controlled using metal oxides.^51^^–^^53^^,^^60^^,^^61^^,^^143^^,^^145^^,^^218^ Absorbers in PAI phantoms are usually a carbon-black variant (Sec. 3.3.2), though dyes and pigments (Sec. 3.3.4) have been used in some cases, especially for spectral matching.^52^^,^^53^^,^^61^^,^^160^^,^^218^ Importantly, optical additions should be selected such that they have a minimal impact on the acoustic properties of the phantom.

The primary acoustic properties of consideration when designing PAI phantoms include the speed of sound and acoustic attenuation. The speed of sound is mostly dependent on the bulk material and should be tailored to the specific PAI application and tissue of interest.^51^^,^^53^^,^^61^^,^^71^^,^^143^ The typical range for speed of sound in soft tissue is 1430 to 1550  m/s, with the lower end of that range encompassing more fatty tissue.^52^^,^^53^ Tuning the speed of sound of the matrix material can be achieved through the addition of softeners and hardeners and blending matrix materials with liquid additives, such as mineral oil, silicone oil, glycerol, and ethylene glycol.^52^^,^^72^^,^^143^^,^^145^^,^^220^ The second significant acoustic property for PAI phantoms, acoustic attenuation, is dependent on the acoustic frequency, which in soft tissue is ∼0.5 to $2\;\mathrm{dB}\;{\mathrm{cm}}^{-1}\;{\mathrm{MHz}}^{-1}$.^52^^,^^71^^,^^143^ Those PAI systems that use relatively high frequencies may require lower acoustic attenuation than the typical range.^53^ Acoustic attenuation can be modulated through matrix material alterations such as the addition of plasticizers and changing the emulsion size.^52^^,^^61^^,^^143^^,^^145^ This property can also be tweaked through the inclusion of acoustic scattering particles such as glass microspheres.^53^^,^^60^^,^^71^^,^^72^

Finally, mechanical properties are also important in PAI, though typically to a lesser extent than the acoustic properties of the phantom material. Density and Young’s modulus are two relatively important mechanical factors, and some PAI applications do require a realistic representation of these properties.^71^^,^^72^ The Grüneisen parameter, which is a measure of thermoelastic efficiency, can also be critical for quantitative PAI, especially for the phantom inclusions as it is typically assumed to be constant in the imaging domain.^143^ However, as this parameter is difficult to measure and has a strong temperature dependence, it is rarely characterized. As such, its importance to general phantom design is currently poorly understood.

#### 4.3.3 Geometry considerations

To provide the necessary contrast for PAI, phantoms require some type of inclusion that has optical contrast compared with the bulk material in which it is embedded or surrounded. Photoacoustic phantoms may be patterned, embedded with molded inclusions of simple geometries such as spheres or tubes with flow channels for blood-mimicking fluid to assess one or more performance metrics.^22^^,^^51^^–^^53^^,^^60^^,^^61^^,^^143^^,^^145^^,^^218^ PAI phantoms may contain more anatomically complex structures or inclusions, such as distinct tissue-mimicking layers, 2D printed biomimicking structures [Fig. 2(d)], or 3D-printed geometries enabled by advanced imaging techniques.^16^^,^^20^^,^^71^^,^^72^^,^^160^ The IPASC has not currently published recommendations on standards for phantom geometry.

**Table 5:** Selected publications focusing on the development of optical phantoms for photoacoustic modalities over the previous 5 years (2020 to 2025).

Authors	Year	Matrix material	Absorber(s)	Scatterer(s)	Geometry	Other	Reference
Dreher et al.	2025	Copolymer-in-oil	Inorganic pigments and dyes	${\mathrm{TiO}}_{2}$	Anatomical	Combination of absorbers to match different blood oxygenation states	186
Xu et al.	2025	Agar hydrogel	India ink	${\mathrm{TiO}}_{2}$	Anatomical	Microvasculature created using loofah fibers	269
Gröhl et al.	2024	Copolymer-in-oil	Nigrosin	${\mathrm{TiO}}_{2}$	Patterned	Paired with digital twins	218
Khodaverdi et al.	2024	Copolymer-in-oil	Inorganic pigments and carbon black	${\mathrm{TiO}}_{2}$	Homogeneous and patterned	—	270
Xu et al.	2024	UV resin and agar hydrogel	Hemoglobin and mineral oil	Intralipid	Anatomical	Made to mimic microstructure of bone	271
Grasso et al.	2023	Copolymer-in-oil and UV resin	Inorganic pigments and carbon black	—	Anatomical	Reconstruction of a morphologically realistic mouse	20
Morsink et al.	2023	Polyurethane	Carbon black	—	Patterned and anatomical	Made using an office laser printer	16
Vogt et al.	2023	Polyvinyl chloride-plastisol	Nigrosin, India ink, and blood	${\mathrm{TiO}}_{2}$	Layered phantom with inclusions	—	145
Yim et al.	2023	Gelatin hydrogel	Synthetic mMelanin	${\mathrm{TiO}}_{2}$	Layered	3D bioprinting of gelatin methacryloyl hydrogels	160
Hsu et al.	2022	Polyacrylamide hydrogel	Nigrosin, gold nanoparticles, tungsten, and blood	${\mathrm{TiO}}_{2}$	Patterned phantoms with inclusions	—	272
Nguyen et al.	2022	Silk hydrogel	Inorganic dye and carbon black	—	Phantom with inclusions	—	273
Palma-Chavez et al.	2022	Polyacrylamide hydrogel	India ink and nigrosin	${\mathrm{TiO}}_{2}$	Phantom with inclusions	—	52
Dantuma et al.	2021	Polyvinyl chloride-plastisol	Carbon black and blood	${\mathrm{TiO}}_{2}$	Anatomical	—	274
Hacker et al.	2021	Copolymer-in-oil	Nigrosin and inorganic pigments	${\mathrm{TiO}}_{2}$	Homogeneous and patterned	—	39
Hariri et al.	2021	Polyacrylamide hydrogel	India ink and carbon black	${\mathrm{TiO}}_{2}$	Phantom with inclusions	Glass beads for controlled acoustic backscattering	53

### 4.4 Optical Coherence Tomography

Common uses of optical coherence tomography (OCT) phantoms include system characterization and validation, performance testing, and validation of novel OCT processing and analysis methods.^21^^,^^43^^,^^44^^,^^73^^,^^74^^,^^222^^–^^224^ The numerous OCT configurations pose additional constraints in phantom design that are specific to that instrument or method and may not apply broadly to all types of OCT. In general, the most important optical aspect of OCT phantoms is mimicking the backscattering behavior of the tissue of interest. However, with OCT providing detailed tomographic imaging of structured tissues, the ability to create accurate representations of thin tissue layers and geometrically complex structures often becomes the most important phantom feature.

#### 4.4.1 Material requirements

The main material requirements of OCT phantoms include the need to match tissue refractive index and light attenuation, the ability to form distinct layers with their own refractive indices, and, in some cases, form intricate, anatomical structures.^21^^,^^43^^,^^44^^,^^222^^,^^225^^,^^226^ Common bulk matrix materials for OCT phantoms include hydrogels (Sec. 3.1.2), with a rising focus on fibrin-based phantoms, silicones (Sec. 3.1.3), and resins (Sec. 3.1.4).^12^^,^^21^^,^^43^^,^^44^^,^^73^^,^^74^^,^^225^ As overall light attenuation is the primary optical feature of interest, absorbing agents are not necessary for OCT phantoms, though carbon black variants (Sec. 3.3.2) are sometimes included.^44^ Scattering agents are typically metal oxides (Sec. 3.2.3), with ${\mathrm{TiO}}_{2}$ being the most common material used.^12^^,^^43^^,^^44^^,^^74^^,^^225^ Each OCT technique has its own material constraints for phantoms. For example, OCT angiography phantoms require fluid flow through the phantom, which is typically comprised of Intralipid.^21^ More technique-specific examples include optical coherence elastography, which requires tissue-like mechanical and elastic properties, and polarization-sensitive OCT, which requires polarization properties such as birefringence.^3^^,^^225^^,^^227^

#### 4.4.2 Geometry considerations

Although homogeneous phantoms can be used to characterize system and optical performance, most OCT applications involve contrast between layers and structures. Thus, most phantoms used for validating novel OCT applications and processing techniques include multiple layers with accurate anatomical thicknesses. The major approaches for achieving these thin layers include spin-coating, airbrushing, and photocuring-based 3D printing techniques.^21^^,^^43^^,^^44^^,^^62^^,^^128^^,^^222^^,^^225^ When more complex anatomical structures, such as embedded vasculature, are desired, they are often manufactured using microfluidic channels.^21^^,^^62^ For ophthalmic OCT phantoms, accurate size, curvature, and lens systems that are representative of the human eye are often desired for system evaluation. These representations have primarily been achieved using prosthetic or artificial eyes as part of the phantom construction.^43^^,^^44^^,^^74^

**Table 6:** Selected publications focusing on the development of optical phantoms for OCT over the previous 5 years (2020 to 2025).

Authors	Year	Matrix material	Absorber(s)	Scatterer(s)	Geometry	Other	Reference
Chavez et al.	2025	Silicone and agar hydrogel	—	${\mathrm{TiO}}_{2}$	Phantom with inclusions	—	207
Fitzgerald et al.	2025	Thermoplastic	—	—	Patterned	Optically smooth microstructures	275
Lee et al.	2025	Silicone	Inorganic pigment	${\mathrm{TiO}}_{2}$ and intralipid	Anatomical	—	209
Navaeipour et al.	2025	Silicone	—	${\mathrm{TiO}}_{2}$	Anatomical	—	276
Bright et al.	2024	Gelatin and agar hydrogels	—	${\mathrm{TiO}}_{2}$ and intralipid	Layered with inclusions	—	277
Zhao et al.	2024	Silicone	—	${\mathrm{TiO}}_{2}$ and intralipid	Anatomical	Accurate retinal layers achieved using spin coating	278
Chang et al.	2022	Silicone	—	${\mathrm{TiO}}_{2}$	Homogeneous and layered	Stretch-induced birefringence	225
Kulmaganbetov et al.	2022	Gelatin hydrogel	—	Polystyrene microspheres	Homogeneous	—	223
Huang et al.	2021	Silicone	—	Gold microspheres	Homogeneous		279
Wang et al.	2021	Silicone	—	Polystyrene microspheres	Homogeneous	Retinal phantom incorporated into a full model eye	280
Zulina et al.	2021	Silicone	—	${\mathrm{TiO}}_{2}$ and glycerol	Layered and anatomical	—	73
Lamont et al.	2020	UV resin and thermoplastic	—	${\mathrm{TiO}}_{2}$	Anatomical	Direct laser writing to mimic retinal cones	62

### 4.5 Raman Spectroscopy

Optical phantoms for Raman spectroscopy (RS) have been developed for various purposes and for different applications. One of the most common uses is to characterize different performance metrics of Raman systems, including the depth specificity achieved by an optical probe and the signal-to-noise ratio of the spectrograph.^29^^,^^40^^,^^54^^,^^147^^,^^228^^–^^231^ Phantom studies have also been used to determine reference spectra for a target molecule in a biological setting and to perform spectral calibration.^41^^,^^45^^,^^92^^,^^166^^,^^193^^,^^232^^,^^233^ Furthermore, phantoms have been used to characterize the impact of physiological variables on acquired Raman spectra. For example, phantoms have been used to evaluate how different tissue layer thicknesses, skin tones, spatial heterogeneities, and optical properties affect the measured Raman spectra.^40^^,^^41^^,^^45^^,^^54^^,^^92^^,^^111^^,^^112^^,^^148^^,^^230^^,^^233^^,^^234^ Due to the biochemical specificity of the measurements, Raman phantoms are typically designed to approximate the biochemical composition of tissue first and match the optical characteristics second, if at all. Because of this, it is critical to understand that almost all materials used in a phantom will have a distinct Raman spectrum that can affect the overall spectrum of the phantom. This makes the selection of materials used for the matrix, absorbers, and scatterers challenging. Determining the right balance between these aspects is often the largest hurdle when creating a Raman phantom.

#### 4.5.1 Optical property considerations

Similar to fluorescence modalities, Raman phantoms should ideally match tissue optical properties at both the laser excitation wavelength and the inelastically scattered wavelength ranges. Typically, only the excitation wavelength is targeted, assuming inelastic scattering is similar to elastic scattering.^40^^,^^45^^,^^54^^,^^111^^,^^148^^,^^166^^,^^193^^,^^228^^,^^230^^,^^234^ However, there has been some success in approximating the optical performance across the excitation and emission wavelength ranges when using NIR light.^112^ When a specific biochemical analyte is added to the matrix, its inclusion will significantly alter either the absorption or scattering coefficients, requiring a more thorough characterization to maintain tissue-mimicking optical performance.^112^^,^^193^ However, some use cases opt to ignore one or more optical properties of the phantom in favor of matching the biochemical composition of the tissue or analyte of interest.^29^^,^^41^^,^^92^^,^^147^^,^^166^^,^^198^^,^^228^^,^^229^^,^^232^

#### 4.5.2 Material requirements

As the goal of Raman phantoms is to mimic the Raman spectra and, therefore, the biochemical composition of the tissue of interest, an organic matrix is typically required, making common candidates include liquid (Sec. 3.1.1) and hydrogel (Sec. 3.1.2) phantoms.^40^^,^^41^^,^^45^^,^^54^^,^^92^^,^^111^^,^^112^^,^^147^^,^^148^^,^^193^^,^^198^^,^^228^^,^^230^^–^^232^^,^^234^ However, when the target of interest is an exogenous material that is a strong Raman scatterer, silicones (Sec. 3.1.3) and resins have been used successfully to enable longer shelf-lives.^29^^,^^166^^,^^229^^,^^233^ When a specific chemical component is targeted in the study, phantoms can be used to examine the effect of different concentrations on the Raman spectra of that material. Some common examples of Raman scatterers of interest include water, glucose, lipids, and exogenous agents in soft-tissue phantoms, and hydroxyapatite in bone phantoms.^40^^,^^41^^,^^45^^,^^54^^,^^92^^,^^111^^,^^112^^,^^148^^,^^166^^,^^193^^,^^230^^,^^232^^–^^234^

Biological absorbers such as hemoglobin (Sec. 3.3.3) and melanin (Sec. 3.3.1) are ideal for tissue-mimicking applications in Raman phantoms as well.^40^^,^^45^^,^^111^ Carbon black and India ink (Sec. 3.3.2) have been utilized in low concentrations without significantly altering the Raman spectra in cases where longer stability of the phantom is required.^45^^,^^54^^,^^112^^,^^148^^,^^230^^,^^234^ Other dyes and pigments (Sec. 3.3.4) are rarely used, however, as they typically have a strong Raman signature. For scattering, lipid-based scatterers (Sec. 3.2.1) are typically used due to their organic nature, although metal oxides (Sec. 3.2.3) have also been used in some applications.^40^^,^^45^^,^^54^^,^^111^^,^^112^^,^^147^^,^^148^^,^^166^^,^^193^^,^^230^^,^^233^^,^^234^ However, high concentrations of metal oxides can provide a significant nonbiological RS signal, impacting the ultimate performance of the phantom.^235^ Beyond purely synthetic phantoms, the use of animal tissue, often in combination with synthetic phantom materials, is commonly used to mimic the range of biological Raman spectra, though these models fall outside the scope of this review.^7^ For all Raman phantoms, one should avoid materials that have strong Raman features in the spectral band of the Raman signal that is being measured to ensure that the target can be accurately measured.

#### 4.5.3 Geometry considerations

Homogeneous phantoms are sufficient for many use cases in RS, such as characterizing system performance and predicting spectral behavior.^41^^,^^92^^,^^112^^,^^147^^,^^148^^,^^166^^,^^193^^,^^232^^,^^234^ However, modeling biological RS often requires the use of a layered phantom or an inclusion to match the micro-anatomy of the tissue of interest.^29^^,^^40^^,^^45^^,^^198^^,^^228^^–^^231^^,^^233^ Layers are often used to match the depth at which a physiological change is occurring or to better approximate the optical properties of layered tissues such as the skin, with melanin localized to the epidermal layer.^40^^,^^45^^,^^228^^,^^231^^,^^233^ In some of the most complex cases, CT scans have been used to create direct replicas of anthropomorphic structures.^111^^,^^198^

**Table 7:** Selected publications focusing on the development of optical phantoms for Raman spectroscopy over the previous 5 years (2020 to 2025).

Authors	Year	Matrix material	Absorber(s)	Scatterer(s)	Geometry	Other	Reference
Guo et al.	2024	Water	Water and sunflower oil	Sunflower oil	Homogeneous	—	193
Jayasankar et al.	2024	Silicone	FAD and NADH	Glycerol	Patterned and layered	Addition of hydroxyapatite	187
Raj et al.	2024	Agar hydrogel	India ink and blood	Intralipid	Patterned, layered, and anatomical	Raman detection of glucose	45
Gautam et al.	2023	Gelatin hydrogel	India ink	Intralipid	Homogeneous	Varied concentration of water and hydroxyapatite	112
LaLone et al.	2023	Gelatin hydrogel	—	—	Homogeneous	Controlled biochemical composition	194
Xie et al.	2023	Agar hydrogel	—	—	Phantom with inclusions	Phantom embedded into tissue	195
Guo et al.	2022	Agar hydrogel	—	Duck fat	Homogeneous	—	196
Fales et al.	2020	UV resin and water	—	${\mathrm{TiO}}_{2}$	Phantom with inclusions	—	197
Sadura et al.	2021	Silicone	—	Glycerin	Patterned	—	166
Shams et al.	2020	Gelatin hydrogel	—	Flour	Anatomical with inclusions	Acetaminophen used as a contrast agent	198

## 5 Confounding Factors and Other Practical Considerations

Although great strides have been made to advance the capabilities of optical phantoms, there are a number of challenges that may arise throughout the manufacturing and utilization processes that are important to be aware of. On the manufacturing side, the resolution of these challenges typically revolves around experience and general know-how that is either developed firsthand or is passed directly from a peer or advisor. Unfortunately, only a few published works describe their methodologies in sufficient detail to address these challenges. Well-reported phantom publications should include sufficiently detailed information on the materials used and fabrication techniques in order for others to easily replicate their work.^15^^,^^39^^,^^129^

### 5.1 Homogeneity and Manufacturing

One of the most common of these challenges pertains to achieving sufficient homogenization of the added scatterers, particularly when using metal oxides or microspheres. When using a concentration of scatterers that best mimics tissue, the material will become substantially white and opaque, as expected. This appearance can serve to hide regions of differing concentrations and larger clumps of material from the eye. To enable increased visible contrast during manufacturing, it can be beneficial to include at least one of the absorbers with the scatterers, with darker or black absorbers working best to highlight regions of clumped scattering agents. The degree of the issue is dependent on the type of scatterer used, with formulations that include stabilizers and emulsifiers tending to be easier to incorporate. Similar aggregation and clumping can also occur when using certain absorbers. Most commonly, this is observed with biological chromophores, with both melanin and hemoglobin requiring specific care or solvents to prevent aggregates from forming and ensure optical homogeneity. In all cases, sonication is recommended as a final step for homogenization as it can affect the distribution of the particles to a finer degree as compared with manual mixing.

Sonication is ideally performed as late in the manufacturing process as possible, without interfering with the solidification process, to counter the related issue of scatterer settling. The majority of the common scatterers used in phantoms have a higher density than the matrix material, so when a phantom in a nonsolid state sits for an appreciable amount of time while waiting to be used or during curing, particles will sink and settle to the bottom, disrupting the homogeneity of the material inclusions. Although this can occur in liquid phantoms, repeated agitation can bring the phantom back to working order, minimizing the potential impact. Most solid phantoms are made as a liquid that then solidifies into shape, providing an opportunity for the scatterers to settle before solidification is complete. This is particularly common for both silicone and resin phantoms as the cure time can last from hours to days. It may be beneficial to cure such phantoms at a higher temperature, which can reduce the curing time to <30 min. However, as the curing process for silicone, as well as resins, is exothermic, providing external heat may cause the phantom to reach a temperature that alters the properties of the material.

Although shortening the solidification time helps to prevent the scatterers from settling, it also increases the likelihood of trapping air within the phantom. These trapped, often microscopic, air bubbles increase the overall scattering of the phantom in an uncontrollable manner, leading to poor phantom performance. To mitigate this loss of performance, a number of degassing approaches are available depending on the material type. For resins and silicones, the use of a vacuum chamber is the most common approach, with phantoms being placed within and kept at a near vacuum for 5 to 30 min to remove any trapped air. When water or other volatile compounds are present, however, they will also be removed through this process. As such, hydrogel phantoms often utilize ultrasonic degassing due to their aqueous base. In addition, although less common, pressure pots can also be used to reduce the effect of air bubbles on the final scattering coefficient by compressing the trapped air into inclusions with a negligible size and waiting until the material has solidified. Finding the right balance between taking the time to degas a phantom without letting any scatters settle often needs to be optimized for each new type of phantom made.

With the development of advanced manufacturing techniques in recent years, new challenges have also arisen due to the complex nature of these techniques. When using spin coating techniques to achieve very thin layers, the process is heavily dependent on the composition of the phantom and the resulting viscosity. When the concentration of additives used in the matrix material is changed, so are the viscoelastic properties, and thus, a new rotational speed and time may be required to achieve the desired thickness. Similarly, when using photopolymerization-based 3D printing, the addition of different absorbers and scatterers to the precursor material may impact the efficiency of the UV curing process, absorbing or dispersing the light before it can be absorbed by the resin. This is particularly true for some metal oxides, such as ${\mathrm{TiO}}_{2}$ and ZnO, as both of these highly scattering materials also have strong UV absorption. This may necessitate using minimum thickness layers and longer UV exposure times to ensure that sufficient photopolymerization is achieved.

### 5.2 Computational Models

When determining and characterizing the optical properties of a phantom, or any other material, an inverse model light propagation model is generally used to extract the absorption and (reduced) scattering coefficients from a set of physical measurements. Thus, a major confounding factor in using phantoms is related to the associated models and the assumptions made in those models. Whether the utilized model is based on adding-doubling, Monte Carlo, or the diffusion approximation, there are a number of implicit assumptions made in these models that will result in inaccuracies in the extracted or calculated optical properties when violated.^236^^–^^238^ The first is the assumption that the measurements do not contain any fluorescence or other luminescence-based secondary signals in the measurements. As most systems used to measure optical properties rely on wavelength selectivity on the input or the output, but not both, measurements can easily be contaminated by contributions from these secondary luminescence sources, resulting in an overestimation of scattering and an underestimation of absorption.^205^ The optical properties of phantoms with an added fluorophore can be approximated based on measurements made in the absence of the fluorophore. However, many phantom materials, including commonly used absorbers, scatterers, and matrix materials, are known to be fluorescent, especially when illuminated with blue and near-UV wavelengths. In such cases, accurate optical properties can only be achieved using precise optical instrumentation where wavelength selectivity is maintained for both the illumination and detection.

Many of these models break down when absorption is too high relative to the reduced scattering, such that any collected signal no longer conforms to the assumptions of the diffusion approximation ($\mu_{s}^{\prime }\gg \mu_{a}$). Unless the incident light source is diffuse, proper use of diffusion approximation-based models requires the collimated or focused light to undergo multiple scattering events before being detected. This is an issue for almost all light transport models as the default assumption is to approximate the angle-dependent scattering behavior, or scattering phase function, of light with the Henyey–Greenstein function, which has been shown to underestimate the true backscattering of a sample.^239^ As the absorption increases, the proportion of detected photons that have undergone multiple scattering events decreases, resulting in a large proportion of the signal coming from sub-diffuse photons. Because of this, any measurement taken will have an increased dependence on the true scattering phase function, resulting in current models breaking down and producing inaccurate optical properties. While there has been some success shown using a multistage processing technique to recover accurate optical properties at wavelengths where the sample material has high absorption peaks, it cannot be applied to all cases.^15^^,^^240^^,^^241^ For improved optical property estimation, more specific scattering phase functions need to be incorporated, or the use of more robust analytical models will be required.^242^ Issues with sub-diffuse measurements can also occur from different measurement geometries, which can result in unexpected optical behavior for a system-under-test when the optical properties are extracted using a diffusion method. The main modalities where these issues have been observed are high spatial frequency SFDI and single-fiber reflectance, both of which are diffuse optical modalities.^239^^,^^243^^–^^248^ In both of these cases, all, or some portion, of the measured signal is characterized by severe spatial limitations in the light collection geometry, which limits the inclusion of diffuse photons. As such, using, predicting, and modeling their behavior for a given set of optical properties can become dependent on incorporating a more robust scattering phase function approximation. How, and to what degree, sub-diffuse photons impact other modalities and measurement geometries is still an ongoing area of investigation that users need to be aware of before designing new phantom characterization methodologies.

Finally, scattering phase function and anisotropy are not the only optical characteristics that can result in a phantom performing differently than expected. When making a diffuse reflectance phantom that accurately mimics the reflectance of tissue, directly matching the absorption and scattering coefficients may not be the best approach when using silicone, resin, or plastic matrix materials. This is because the refractive index of the matrix material significantly impacts the measured diffuse reflectance for the same optical properties. With most biological tissues having a refractive index in the range of 1.35 to 1.45, many inorganic matrices have refractive indices that are significantly higher, with many falling within the 1.5 to 1.6 range. These refractive indices need to be accurately accounted for in the model so that phantoms can be designed with specific diffuse reflectance values. Otherwise, one should keep in mind that the desired reflectance measurements will vary from those at the targeted tissue, despite the appropriate additions in the phantom design.

### 5.3 Recommendations for Standardization of Phantom Reporting

Standardization in optical phantom design, especially approaches within a given modality, is a necessary direction to enhance the standardization and reproducibility of optical systems and thus the clinical impact of optical techniques, as introduced in Sec. 1.1. In this review, we aim to centralize practical information on the state of the art in the optical phantom space over the past two decades and capture the beginning stage of standardization efforts in the field. As demonstrated in Sec. 4, the considerations for each optical modality are unique and thus require specialized effort from the leaders in a given technique to form a consensus on best practices for phantom design and system validation. The wider field, however, can adopt a basic standard reporting scheme to ensure that the necessary information is being provided for replication of the phantom, and therefore, the validation and/or testing completed with that phantom.

When a phantom design is being reported, the following information should always be included in the publication:

1. The direct material sources for all phantom components (matrix material, absorbers, scatterers), including the manufacturer, product number, and batch number (if applicable)
2. The concentrations of all components used in the phantom
3. The physical dimensions of the phantom(s) being fabricated
4. A detailed manufacturing methodology, including the order of component addition; mixing durations and methods; mold geometry and fabrication methods (if any); degassing protocols; and any additional techniques to ensure an optimal outcome (e.g., those described in Sec. 5.1)
5. Applicable methods of optical, biochemical, mechanical, or other physical characterizations of all components used
6. Detailed characterization of the phantom, potentially including a comparison with ground truth or expected measurements (e.g., optical properties, reflectance, or other recorded signals)
7. Details of the models used to extract optical properties from the physical measurements made.

In addition to these basic pieces of information, authors should report any characteristics and metrics for their phantom and the modality being targeted. Examples may include fluorescence behavior, Raman spectra, or thermal and acoustic information. To further enhance the standardization of phantom design and testing across optical systems, it is also best practice to make the phantom recipe, fabrication protocol, and any algorithms used publicly available, whether it be in the paper itself, in supplemental information, or in a repository such as GitHub that is referenced within the paper.

For the case of manuscripts using previously published phantoms and fabrication protocols, especially when optical characterization may not be feasible for the lab, or the protocol was previously described with sufficient detail for replication of the phantom, items 1 to 4 in the list above may be sufficient for minimum reporting. However, ideally, components used in phantoms should always be routinely characterized before use due to batch-to-batch differences in components, whether they are biological or inorganic, and no matter where they are sourced from, and should always be validated before being employed to test an instrument or system.

## 6 Phantom Application Example: Understanding and Accounting for Skin Pigmentation

The ubiquitous use of pulse oximeters, not only in hospitals and other clinical settings, but also as home care monitoring devices, during the COVID-19 pandemic is a recent example that highlights the significance of skin tone in the accuracy of pulse oximeters and, more generally, in the field of biophotonics and optical technologies. The COVID-19 pandemic brought into sharp focus critical disparities in pulse oximeter accuracy across different skin tones, with studies demonstrating systematic overestimation of blood oxygen saturation (${\mathrm{SpO}}_{2}$) in patients with darker skin pigmentation.^249^^–^^252^ These measurement biases, which ranged from 2% to 5% in some patient cohorts, carried significant clinical implications as they could mask occult hypoxemia in Black and Hispanic patients, potentially delaying life-saving interventions such as supplemental oxygen therapy or mechanical ventilation.^253^ The U.S. Food and Drug Administration (FDA) responded to this issue in the midst of the COVID-19 pandemic, releasing a brief acknowledging concerns about the accuracy of pulse oximeters for darker-skinned users in 2021, and has since released research studies, discussion papers, and updated guidance about pulse oximeters directly pertaining to the impact of skin tone on these devices.^254^ Traditional pulse oximeters operate on the principle of differential absorption of red (660 nm) and infrared (940 nm) light to calculate the ratio of oxygenated to deoxygenated hemoglobin, with the underlying assumption that interference from melanin absorption in the epidermis is minimal or negligible.^184^ However, melanin exhibits strong, wavelength-dependent absorption characteristics that preferentially attenuate shorter wavelengths (red light) more than infrared light. Analysis of Monte Carlo–simulated reflectance photoplethysmography (PPG) signals revealed that with increasing skin pigmentation, systolic intensity decreased by 3% to 4% at the red wavelength (660 nm), whereas the impact at the infrared wavelength (940 nm) remained minimal at <0.2%.^253^ These models explain the clinical results, demonstrating how the wavelength-dependent absorption of melanin directly affects the ratio calculations that form the basis of pulse oximetry measurements, therefore artificially inflating the calculated ${\mathrm{SpO}}_{2}$ values in individuals with higher melanin content.

Of course, melanin has long been considered a prominent chromophore in the skin.^159^ Melanin and other broadband absorbers have been used in phantoms for decades—largely for the modeling of optical approaches for dermatological applications.^49^^,^^255^ Skin pigmentation has, in fact, been specifically mimicked in phantom studies and Monte Carlo models prior to the COVID-19 pandemic, mostly in spectroscopy systems.^256^^,^^257^ Several studies even corrected for skin pigmentation in diffuse reflectance spectroscopy measurements in the early 2000s.^48^^,^^258^ Despite these early correction techniques and general acceptance that skin pigmentation impacts light propagation through skin, it was not widespread practice to carry out these calibration studies in preparation for clinical deployment of novel optical systems. For the pulse oximetry application in particular, the difficult task of controlling oxygen saturation in a phantom has limited its application, with calibration commonly being carried out with human subjects instead. Since the COVID-19 pandemic shed light on the insufficient pulse oximeter calibration for people with dark skin pigmentation, there have been increased interest in understanding the skin tone impact on these devices, with many groups developing advanced phantoms, Monte Carlo models, or both, for these investigations.^124^^,^^184^^,^^253^^,^^259^^–^^261^

In addition to the increased frequency of these investigations, novel designs and methods have been employed for dynamic phantom designs that enable a range of properties, such as skin tone or oxygenation, to be controlled in phantom experiments. Several notable studies have attempted to fill this void in recent years. For example, a modular phantom design with stackable silicone layers representing skin layers was developed in 2024.^262^ This modular design allows many patient factors, such as skin tone and adipose thickness, to be controlled to characterize their impact on optical measurements. Another set of phantoms, published in 2023, combined inorganic pigments and common scatterers to accurately match the optical properties of various tissues, including pale and melanistic skin, across a broad spectral range [Fig. 2(c)].^15^ Furthermore, the attention drawn to this issue expanded beyond pulse oximetry to other optical techniques that make measurements of or through the skin, with many groups investigating skin tone impacts with phantom studies for NIRS, PAI, SFDI, OCT, and more.^262^^–^^265^

In this section, we will discuss two recent phantom designs to demonstrate how considerations in phantom geometry, component selection, modality-specific criteria, and practical factors that allow us to extract physiological information from physical measurements can be applied to phantom designs for the investigation of skin tone impacts on optical techniques.

### 6.1 Application #1: Morphologically Compatible Finger Phantoms for Pulse Oximetry Performance Testing

Published in 2025, Bhusal et al. designed a finger phantom that generates physiologically realistic photoplethysmography (PPG) signals for the characterization of the impact of patient factors on pulse oximeters.^184^ Due to the importance of compatibility with the finger clip pulse oximeter design for clinically relevant testing, an anatomical finger phantom was created. A digital model of a hand was used to create a 3D-printed mold of the distal phalanx to cure the phantom material in an anatomically realistic shape. As the goal of this phantom was to serve as a reproducible, long-lasting standard for pulse oximeter testing, the design balanced realism with fabrication simplicity. Although anatomical realism was necessary for compatibility with pulse oximeter clips, realism of the internal vasculature structure was not necessary to generate a biorelevant PPG signal. Therefore, a simple U-shape was employed for the flow channel embedded into the phantom. A water-soluble material was added onto the existing finger mold with an extrusion printer to form the U-shaped vessel. A key factor in the matrix material selection for this phantom is the need for biologically realistic mechanical properties, such as elasticity, shore hardness, and compliance of the vessel channel. Due to silicone’s long-term stability and biomimicking mechanical properties, this study evaluated and mechanically characterized two silicone-based materials, PDMS (SYLGARD-184) and EcoFlex, as potential matrix materials for this design. Although biomimicking elastic modulus values were able to be achieved with both materials, surface adhesion of the PDMS phantom made it difficult to work with, leading to the Ecoflex phantom being used in the PPG measurements.

Beyond mechanical properties, the components were selected to match the optical properties needed for the desired use case of the phantom. Scattering in the silicone phantom was mimicked with ${\mathrm{TiO}}_{2}$, and no absorbers were added to the bulk silicone material. Instead, the absorption contributions came from localized broadband absorption by epidermis-mimicking layers with nigrosine. Four layers were created with varying concentrations of nigrosine to simulate different melanosome volume fractions, correlating with skin tones. The fluid in the flow channel contributed a blood-like absorption signature. In this study, the intent was to demonstrate the feasibility of biologically accurate PPG waveforms; thus, controlled oxygenation or spectral peaks associated with blood absorption were not required in this design. Therefore, the blood-mimicking fluid employed in this study mimicked blood-like absorption of moderately saturated blood at the two wavelengths used by pulse oximeters, 660 and 940 nm, through a certain concentration of nigrosine. The phantom material’s optical properties were validated through double-integrating sphere measurements and inverse-adding doubling calculations. This blood-mimicking fluid was pumped through the flow channel with a pulsatile fluid system to generate pulse waves, and the PPG signals were recorded with the various epidermis-mimicking layers. This comprehensive method for mimicking the physiological phenomenon that pulse oximeters extract information from demonstrates the potential for a reproducible and robust standard for testing.

Importantly, the phantom system successfully generated measurements in several clinical pulse oximeters, and variations in photoplethysmography waveform characteristics could be systematically produced by varying the simulated epidermal melanin content. The development of tissue-simulating phantoms representing different melanin concentrations offers a practical benchtop platform for pre-market device assessment, potentially reducing the need for extensive human subject recruitment across diverse populations while ensuring equitable performance across all skin tones.

### 6.2 Application #2: Beyond Pulse Oximetry: Multilayer Flow Circuit Phantom to Test Skin Tone Impacts on Photoacoustic Imaging

Validation studies extending beyond pulse oximetry to photoacoustic imaging have revealed both the generalizability of skin tone effects across optical modalities and the varying success of calibration strategies in different measurement contexts. Photoacoustic imaging experiments utilizing computational skin models, two-layer melanin-containing tissue phantoms simulating epidermal melanin fractions ranging from 1% to 8%, and mice of consistent genetic background but varying pigmentation, consistently demonstrated overestimation of blood oxygen saturation with increasing melanin concentration.^263^

Due to the importance of localized optical contrast to generate relevant PAI signals, two layers and a blood flow channel inclusion with distinct biologically relevant optical properties were incorporated in the design. The broadband absorber, synthetic melanin, was included in a thin, epidermis-mimicking outer layer of the phantom, with multiple phantoms created at varying melanin concentrations to simulate various Fitzpatrick skin types across different measurements. The matrix material selected was agarose as hydrogels are often used to mimic both optical and acoustic properties needed for PAI, and more mechanical robustness compared with a softer gelatin material was required due to the horizontal orientation of the phantom in the measurement geometry. Intralipid was included to match the scattering properties of the skin. Tubing allowed for blood flow through the center of the phantom, in which fresh whole blood from rats was used. Because measurements were taken within a short time of blood extraction and it was kept in a separate compartment from the rest of the phantom, whole blood was able to be used to provide comprehensive, broad-spectrum absorption characteristics. The blood circulation was controlled by a flow circuit that used a peristaltic pump for blood circulation and allowed for controlled deoxygenation through the administration of sodium dithionite, with ground truth oxygen levels taken with oxygen fluorescence quenching needle probes.^263^ The desired optical properties of the epidermis-mimicking layers were validated through double-integrating sphere measurements on thin 2D test phantoms and inverse-adding doubling calculations. In addition, the model used to extract physiological information from physical PAI measurements made with phantoms was also evaluated for impact based on skin tone in Else et al.^263^ Standard practice for oximetry with PAI measurements in PAI involves using linear spectral unmixing to extract chromophore concentrations, in this case, oxy- and deoxy-hemoglobin. However, this technique assumes that the measured signal is a linear combination of chromophores at each pixel, which does not account for the wavelength-dependent behavior of such chromophores. In contrast, learned unmixing methods based on gradient-boosted regression partially mitigated these errors by training on comprehensive datasets spanning different melanin concentrations.^266^ However, even these advanced machine learning approaches demonstrated residual skin tone-dependent effects when predicting lower blood oxygen saturation levels, indicating that computational correction has inherent limitations when the underlying signal quality is compromised by melanin absorption.

### 6.3 Conclusion

Development of robust optical phantoms for system performance characterization and intersystem comparison is necessary in the ongoing development of optical systems for clinical applications. With the FDA initiating an effort to review guidelines for pulse oximeter calibration in 2024, optical phantoms should be considered for their potential to improve the calibration process across melanin concentrations and oxygen concentrations.^124^^,^^160^^,^^254^^,^^267^ Furthermore, as phantoms become more accurate, rigorous characterization of device performance across various patient factors, from skin tone to BMI, is able to be assessed with insights from these models. Continued development in phantom accuracy and efforts to standardize phantoms across groups will enhance applicability toward commercial systems.

Despite these promising recent designs and developments in phantoms, significant limitations remain that must be addressed before phantom-calibrated systems can achieve widespread clinical adoption across all optical sensing modalities and patient populations. Current phantom systems predominantly represent homogeneous or simple two-layer tissue models, whereas actual skin exhibits complex stratified structures with varying optical properties within the epidermis, papillary dermis, reticular dermis, and subcutaneous layers. In addition, the distribution of melanin within melanosomes, the packaging of melanosomes within keratinocytes, and the spatial arrangement of these cells within the epidermis all influence light transport in ways that are challenging to replicate with synthetic materials. Even some of the most complex phantoms that model random distribution of melanin clumps using 3D-bioprinting fabrication methods face inherent challenges in replicating the full complexity of human tissue, including difficulties matching the refractive indices of heterogeneous tissues in the skin and limitations in achieving accurate penetration depths.^160^ Furthermore, the darkest and lightest extremes of skin tone are difficult to physically replicate with phantoms, limiting the validation studies that can be performed with phantoms.^263^ This gap is particularly concerning for the darkest skin tones, equivalent to Fitzpatrick scale type VI, where melanin concentrations are highest and measurement biases would be expected to be most severe. Future work must prioritize active recruitment efforts to expand validation across the complete spectrum of human skin tones in tandem with the development of more sophisticated multilayer phantoms with anatomically accurate optical properties to establish regulatory pathways for phantom-calibrated devices that can deliver equitable performance for all patients regardless of skin pigmentation.

## 7 Discussion and Future Directions

The development of tissue-mimicking optical phantoms has significantly advanced over the past two decades, fueled by increasing demands for rigorous validation and calibration of optical technologies. As reviewed in this paper, substantial progress has been made in diversifying matrix materials and utilizing different classes of scatterers and absorbers to replicate a range of tissue types and optical phenomena. However, despite these innovations, the field continues to face persistent challenges that must be addressed to fully realize the potential of phantoms as standardized, reproducible tools across optical modalities.

One of the most pressing issues remains the lack of standardization across phantom materials, designs, and usage protocols. This variability undermines cross-laboratory reproducibility, inhibits device comparison, and limits the translation of benchtop technologies into clinical practice. Drawing inspiration from efforts such as the IPASC and building on phantom use in other imaging modalities such as MRI and ultrasound, there is a clear need for broader, modality-specific initiatives aimed at developing consensus guidelines on optical phantom fabrication, characterization, and use. Future efforts should also emphasize interoperability and the creation of open-access phantom libraries that include validated optical property datasets.

A second critical area for future research is the inclusion of biologically and demographically relevant features in phantom design. The recent focus on skin-tone-aware phantom development highlights broader issues of bias in biophotonics. Phantoms that replicate the optical properties of melanin-rich tissues across a physiologically relevant range can help address performance disparities in devices such as pulse oximeters. Expanding this concept to include age-, sex-, and disease-specific tissue characteristics could greatly enhance the value of optical systems across diverse populations.

In addition, with advances in 3D printing, microfluidics, and soft robotics, there is an opportunity to develop dynamic, anatomically realistic phantoms that simulate complex biological functions such as blood flow, contrast agent perfusion, or even metabolic activity. These high-fidelity phantoms could bridge the gap between *in vitro* testing and *in vivo* validation, particularly for applications that rely on real-time feedback. However, fabrication techniques of such phantoms are complex and may suffer from challenges in reliable reproducibility.

Finally, computational modeling that extracts optical properties and, by extension, physiological parameters, from the physical measurements of light in its various forms emanating from phantoms, remains an underexploited avenue, particularly in the sub-diffuse regime. Hybrid approaches that pair physical phantoms or digital twins with more accurate computational models of light propagation in tissue or tissue-mimicking phantoms can provide deeper insight into light-tissue interactions and enable more rigorous system optimization. Machine learning approaches represent another sophisticated calibration strategy, employing artificial neural networks and support vector machine regression trained on comprehensive phantom datasets with precisely known optical properties.^263^^,^^266^^,^^268^ Computational modeling with Monte Carlo or machine learning approaches have demonstrated great potential to capture physiological processes that may be difficult to replicate physically in phantom models, such as controlled oxygen saturation in oximetry devices. Thus, they show great potential to be used for standardization and calibration efforts in conjunction with physical phantom models.

In summary, the field of tissue optical phantoms is rich with innovation, yet requires targeted coordination and investment to evolve from a highly customized, research-driven discipline into a robust, standardized, and inclusive component of translational biophotonics.

## Data Availability

Data sharing is not applicable to this article as no new data were created or analyzed.
